# Safety, tolerability and toxicokinetics of the novel mitochondrial drug SUL-138 administered orally to rat and minipig

**DOI:** 10.1016/j.toxrep.2024.03.009

**Published:** 2024-03-19

**Authors:** Daniël H. Swart, Martin de Haan, Jasper Stevens, Rob H. Henning, Sovan Adel, Adrianus C. van der Graaf, Nadir Ulu, Daan J. Touw, Guido Krenning

**Affiliations:** a Department of Clinical Pharmacy and Pharmacology, University of Groningen, University Medical Center Groningen, Hanzeplein 1, Groningen 9713GZ, the Netherlands; b Sulfateq B.V., Admiraal de Ruyterlaan 5, Groningen 9726GN, the Netherlands; c Madeha Management & Consultancy B.V., Eilandseweg 10, Nederhorst den Berg 1394JE, the Netherlands; d Gen İlaç ve Sağlık Ürünleri A.Ş., Mustafa Kemal Mahallesi, 2119.Cad. No:3, Çankaya, Ankara 06520, Turkey; e Department of Pharmaceutical Analysis, Groningen Research Institute of Pharmacy, University of Groningen, Groningen 9713GZ, the Netherlands

**Keywords:** Noncommunicable diseases, 6-chromanols, Drug development, Metabolism, NOAEL, Preclinical toxicology

## Abstract

Noncommunicable Chronic Diseases (NCD) are a socioeconomic burden and considered one of the major health challenges for coming decades. Mitochondrial dysfunction has been implicated mechanistically in their pathophysiology. Therefore, targeting mitochondria holds great promise to improve clinical outcomes in NCDs. SUL-138, an orally bioavailable small molecule efficacious from 0.5 mg/kg, improves mitochondrial function during disease in several preclinical animal models. As preparation for a First-in-Human (FIH) trial, SUL-138 was investigated in 30-day GLP repeated dose toxicity studies in rat and minipig, selected based on their comparability with human metabolism, to determine toxicokinetics, potential toxicity and its reversibility. Rats were allocated to either vehicle, 27, 136 or 682 mg/kg SUL-138 dose groups and minipigs were allocated to either vehicle, 16, 82 or 409 mg/kg. Treatment occurred orally for 30 days followed by a recovery period of 14 days. During these studies clinical observations, toxicokinetic, clinical pathology, necropsy and histopathology evaluations were performed. There was significant systemic exposure to SUL-138 and toxicokinetics was characterized by a rapid absorption and elimination. In the rat, toxicokinetics was dose-proportional and AUC_0-tlast_ ratios in both species indicated that SUL-138 does not accumulate *in vivo*. No treatment-related adverse effects were observed for dose levels up to 136 and 82 mg/kg/day in rat and minipig respectively. In conclusion, these preclinical studies demonstrate that SUL-138 is well tolerated after repeated administration in rat and minipig, with NOAELs of 136 and 82 mg/kg/day, respectively.

## Highlights

• SUL-138 is rapidly absorbed, eliminated and does not accumulate *in vivo.*
• Toxicokinetics of SUL-138 are dose-proportional in the rat.
• C_max_ is dose-proportional and AUC_0-tlast_ is disproportional in the minipig.
• SUL-138 is well tolerated by rat and minipig in GLP toxicology studies.
• Rat and minipig NOAEL are respectively 136 and 82 mg/kg/day.

## Graphical Abstract

**Figure:**
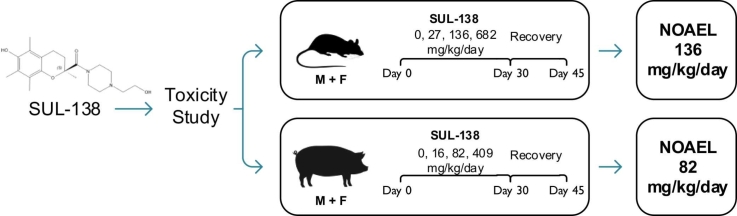

## 1 Introduction

Noncommunicable Chronic Diseases (NCD), such as cardiovascular, kidney, metabolic, neurodegenerative and respiratory disease, represent a burden to society. They are the leading causes of mortality responsible for 74% of all deaths worldwide, equivalent to 41 million people annually [1], [2]. NCDs are associated with tremendous healthcare costs. Economic simulations by the World Economic Forum and the Harvard School of Public Health indicate a cumulative cost of $47 trillion from 2011 to 2030 [3]. The enormous socioeconomic burden of NDCs create an unmet medical need for new therapeutic interventions.

Mitochondrial dysfunction is a common denominator in NCDs and plays a key role in their pathophysiology [4]. Mitochondrial dysfunction is associated with pancreatic β-cell dysfunction and insulin resistance in Type 2 Diabetes (T2D), obesity and cardiometabolic syndrome [5], [6], Chronic Heart Failure (CHF) [7], [8], stroke [7], [8], cancer [9] and Chronic Obstructive Pulmonary Disease (COPD) [10], [11]. In addition, there is accumulating evidence that mitochondrial dysfunction and an imbalance between ATP and ROS play a key role in the etiology of neurodegenerative diseases such as Alzheimer’s and Parkinson’s Disease [12], [13], [14]. Even in acute diseases, such as Acute Kidney Injury (AKI) and sepsis, mitochondrial dysfunction is believed to be the primary risk factor for disease progressing into respectively Chronic Kidney Disease (CKD) and organ failure [15], [16]. Collectively, these data indicate that mitochondria represent an innovative therapeutic target and that improving mitochondrial function during pathology holds great potential for ameliorating disease progression and improving clinical outcomes in NCDs.

6-chromanol compounds specifically target mitochondrial dysfunction during disease by maintaining mitochondrial network integrity and preserving mitochondrial energy production through activation of complex I and IV [17], [18]. Lead compound SUL-138 shows therapeutic efficacy in animal models for Ischemia-Reperfusion AKI (0.5 mg/kg), kidney dysfunction in sepsis (5 mg/kg) and Alzheimer’s Disease (30 mg/kg/day) [18], [19], [20]. Transition of SUL-138 from a preclinical to clinical development stage requires stringent toxicology and toxicokinetic evaluation. Therefore, GLP 30-day toxicity studies in rats and minipigs, species selected based on their comparability with human metabolism and studies designed according European Medicines Agency (EMA) and Food and Drug Administration (FDA) guidance documents, were performed. The aim of the present studies was to determine SUL-138 toxicokinetics, potential toxicity, delayed onset toxicity and reversibility of findings after repeated oral administration. From the study data a No Observed Adverse Effect Level (NOAEL) will be derived which can be used to determine the Maximum Recommended Starting Dose (MRSD) for a First-in-Human (FIH) clinical trial [21].

## 2 Materials and methods

### 2.1 Study drug and formulation

The hydrochloric acid (HCl)-salt of SUL-138 (SUL-238), purity > 98%, was manufactured by Ofichem (Ter Apel, The Netherlands). Dose formulations were prepared weekly by dissolving the required amount of SUL-138 in 0.9% Sodium Chloride (NaCl). The vehicle formulation consisted of 0.9% NaCl. After preparation, formulations were stored at 2 – 8 °C and dose concentrations were confirmed by High Performance Liquid Chromatography (HPLC) analysis.

### 2.2 General toxicity studies

Toxicity studies in rats and minipigs were conducted at European Research Biology Center S.r.l. (ERBC, Pomezia, Italy) in accordance with OECD Good Laboratory Practice (GLP). Male and female Hsd: Sprague Dawley (SD) rats (6 – 7 weeks old, body weight 150 – 190 g) were obtained from Envigo RMS S.r.l. (San Pietro al Natisone (UD), Italy). Drinking water and standard laboratory chow (4 RF 21, Mucedola S.r.l., Via G. Galilei 4, 20019 Settimo Milanese (MI), Italy) were supplied ad libitum. Rats were allocated to four dose groups each composed of three subgroups: main, recovery and toxicokinetic satellite (Table 1). The highest dose was selected based on the Maximum Tolerated Dose (MTD) obtained in a preliminary dose range finding toxicity study (unpublished). SUL-138 was administered once daily by oral gavage (10 mL/kg) for 30 days. After 30 days animals from the main and toxicokinetic subgroup were euthanized and the recovery animals were untreated for a 14-day recovery period to assess reversibility of potential treatment-related effects before being euthanized.

**Table 1:** Study design of the 30-day GLP toxicity study in Sprague Dawley rats with a 14-day recovery period.

Group	Dose SUL-138 (mg/kg/day)	N (M/F)			
	Free base	Main		Recovery	Toxicokinetic
1	Vehicle	10/10		5/5	3/3
2	27	10/10			9/9
3	136	10/10			9/9
4	682	10/10		5/5	9/9
**Evaluations**			**Interval**		
Clinical observations			Once daily and weekly outside home cage		
Body weight			Twice a week		
Food intake			Weekly		
Body temperature			Day 1 and Day 29 at predose, 1, 4 and 8 hours		
Ophthalmoscopy			Before start treatment and at day 24		
Clinical pathology and urinanalysis			End of treatment and at end of recovery		
Toxicokinetics			Day 1: 0.13, 0.25, 0.5, 1, 2, 4, 8 and 24 hours post-dose Day 29: predose, 0.13, 0.25, 0.5, 1, 2, 4, 8 and 24 hours post-dose (n: 3 per sex/dosing group)		

Male and female Göttingen minipigs (4 – 5 months old, body weight 11 – 15 kg) were obtained from Ellegaard Göttingen Minipigs (Dalmose, Denmark). Drinking water was provided ad libitum and each minipig received a daily weighted amount of diet divided in two rations (Altromin 9029, Maintenance diet for minipig, Altromin Spezialfutter GmbH & Co. KG, Im Seelenkamp 20, 32791 Lage, Germany). Minipigs were allocated to four dose groups each composed of two subgroups: main and recovery (Table 2). The highest dose was selected based on the MTD obtained in a preliminary dose range finding toxicity study (unpublished). SUL-138 was administered once daily by oral gavage (10 mL/kg) for 30 days. After 30 days of treatment, animals from the main group were euthanized and the recovery animals were untreated for a 14-day recovery period to assess reversibility of potential treatment-related effects before being euthanized.

**Table 2:** Study design of the 30-day GLP toxicity study in Göttingen minipigs with a 14-day recovery period.

Group	Dose SUL-138 (mg/kg/day)	N (M/F)		
	Free base	Main		Recovery
1	Vehicle	3/3		2/2
2	16	3/3		
3	82	3/3		
4	409	3/3		2/2
**Evaluations**			**Interval**	
Clinical observations			Once daily	
Body weight			Weekly	
Food intake			Daily	
Body temperature			Day 1 and Day 29 at predose, 1, 4 and 8 hours	
Ophthalmoscopy			Before start treatment and at day 29	
Electrocardiography			Before start treatment, day 1 and day 29 at predose and 2 hours post dose	
Clinical pathology and urinanalysis			Before start treatment, end of treatment and end of recovery	
Toxicokinetics			Day 1: 0.13, 0.25, 0.5, 1, 2, 4, 8 and 24 hours post dose Day 29: predose, 0.13, 0.25, 0.5, 1, 2, 4, 8 and 24 hours post dose (n: 3 per sex/dosing group)	

### 2.3 In vivo observations

To evaluate the effects of repeated oral administration of SUL-138 all animals were monitored for clinical signs. In addition, rats were observed outside their home cage in a separate arena to assess responsiveness to different sensory stimuli, grip strength and motor activity. Throughout the studies body weight and food consumption were recorded. Eyes were examined for ocular lesions with an ophthalmoscope following instillation of 0.5% (rat) and 1.0% (minipig) Tropicamide (Visumidriatic®, Visufarma, Rome, Italy). Further, rectal body temperature was measured. Thirdly, electrocardiographic tracings were recorded in all minipigs by using synchronously the three standard limb leads (I, II and III) and the augmented limb leads (aVR, aVL and aVF).

### 2.4 Clinical pathology and urinalysis

Blood and urine samples were obtained from rats and minipigs after overnight deprivation of food and water. Rats received 10 mL/kg of drinking water by oral gavage prior to urine collection to ensure suitability of urine samples for analysis. In rats, blood samples were obtained under isoflurane anesthesia from the vena cava just shortly before necropsy. In minipigs, blood samples were drawn from the jugular vein. All blood samples were collected and analyzed for hematology-, coagulation-, and clinical chemistry parameters. Urinalysis was performed on all urine samples. Selection of parameters for clinical pathology studies were in compliance with scientific consensus and Organization for Economic Cooperation and Development (OECD) guidelines [22], [23], [24]. In more detail the following hematology parameters were evaluated by a Siemens Advia 120: Hematocrit (HCT), Hemoglobin (HGB), Red Blood Cell count (RBC), Reticulocyte count (RET), Mean Red Blood Cell Volume (MCV), Mean Corpuscular Hemoglobin (MCH), Mean Corpuscular Hemoglobin Concentration (MCHC), White Blood Cell count (WBC), Neutrophil count (NEU), Lymphocyte count (LYM), Eosinophil count (EOS), Basophil count (BAS), Monocyte count (MON), Large Unstained Cells (LUC) and Platelets (PLT). Coagulation parameters were evaluated by Instrumentation Laboratory ACL Elite PRO: Prothrombin Time (PT) and Activated Partial Thromboplastin Time (APTT). Clinical chemistry parameters were evaluated by a Siemens Advia 1800: Alkaline Phosphatase (ALP), Alanine Aminotransferase (ALT), Aspartate Aminotransferase (AST), Albumin (ALB), Globulin (GLO), Albumin/Globulin ratio (A/G), Urea, Creatinine (CREA), Glucose (GLU), Total Bilirubin (TBIL), total Cholesterol (CHOL), total Protein (PROT), Triglycerides (TRIG) (minipig only), Sodium (Na), Potassium (K), Calcium (Ca) and Chloride (Cl).

### 2.5 Necropsy and histopathology studies

At the end of the study, rats were euthanized under isoflurane anesthesia by exsanguination and minipigs were sedated and euthanized by an intravenous injection of a barbiturate overdose followed by exsanguination. Subsequently, animals were submitted to a detailed macroscopic inspection. Organs were dissected free from fat and weighted before fixation. Fixated biopsies from tissues were dehydrated, embedded in paraffin, sectioned into 4–5 µm thick slices, loaded onto glass slides and stained with haematoxylin and eosin (HE). At first, microscopic evaluation was performed on collected tissues from the rats in the vehicle and high dose main groups. It was later extended to the adrenal glands, kidneys, liver and spleen from the low dose, intermediate dose and recovery groups. For the minipig all collected tissues from the main groups were microscopically examined.

Selection of tissues for pathology studies were in compliance with OECD guidelines [22], [23]. Organ weights were obtained from: adrenal glands, brain, epididymis, gall bladder (minipig only), heart, kidneys, liver, ovaries, pituitary gland, prostate gland (rat only), spleen, testes, thymus, thyroid gland and uterus. The following tissues were microscopically examined: adrenal glands, aorta, bone marrow (from sternum), brain (cerebrum, cerebellum and medulla/pons for the minipig), caecum, coagulating glands (rat only), colon, duodenum, epididymis, eyes, femur with joint, gall bladder (minipig only), harderian glands, heart, ileum, jejunum (including Peyer’s patches), kidneys, liver, lungs, lymph nodes, mammary area, oesophagus, optic nerves, ovaries, oviducts, pancreas, parathyroid glands, pituitary gland, prostate gland, rectum, salivary glands, sciatic nerve, seminal vesicles, skeletal muscle, skin, spinal cord, spleen, stomach, testes, thymus, thyroid gland, tongue, trachea, ureters, urinary bladder, uterus-cervix and vagina.

### 2.6 Toxicokinetics

Blood samples were obtained from the tail vein and jugular vein for rat and minipig respectively, timing depicted in Table 1 and Table 2. Rats were only sampled three times in consideration of animal welfare. Upon collection, blood samples were collected in K_2_EDTA tubes and plasma was obtained through centrifugation at 1000 g for 10 minutes at 2–8 °C. Levels of SUL-138 in plasma were quantified by using a GLP validated LC-MS/MS methods for rat and minipig.

Plasma samples, supplemented with a deuterated internal standard (IS) SUL-138_d5 (Symeres, Groningen, The Netherlands), were mixed thoroughly with acetonitrile and subsequently centrifuged for 10500 g for 10 minutes at 4 °C to precipitate proteins. Supernatant samples containing SUL-138 were submitted to liquid chromatography by using a Shimadzu HPLC apparatus (Shimadzu, Duisburg, Germany) and Kinetex C18 column (100 Å, 2.6 μm, 50 ×4.6 mm) (Phenomenex, Aschaffenburg, Germany). The gradient was composed of solvent A and B, respectively mixtures of acetonitrile/water/ammonium formate (60:40:0.03%) and acetonitrile/water/ammonium formate (95:5:0.03%). Following liquid chromatography MS/MS detection of SUL-138 (*m/z* 205.1) was performed on an API 4000 (AB Sciex, Concord, Canada) with an Ion Spray voltage of 5000 at 500°C. Limit of Detection (LOD) was determined at 0.456 ng/mL and 0.38 ng/mL for rat and minipig respectively. Lower Limit of Quantification (LLOQ) was set at 10.00 ng/mL.

Toxicokinetic non-compartmental analysis was performed in R version 4.2.3 by using the package ncappc version 0.3.0 [25], [26]. Plasma concentrations of SUL-138 below the Lower Limit of Quantification (LLOQ), 2.0% for the rat and 14% for the minipig, were replaced with 0.5 * LLOQ for calculation purposes. Area Under the plasma concentration-time Curve (AUC_0-tlast_) was calculated by using the linear trapezoidal rule. Similarly, AUC_0-∞_ was also calculated by using the linear trapezoidal rule and extrapolated to infinity based on the last measured plasma concentration (C_last_). The terminal half-life was calculated by linear regression of at least the last three data points, excluding C_max_, in the elimination curve. Half-life and associated AUC_0-∞_ were reported if the adjusted correlation coefficient (R^2^ adj) was greater than 0.7. Mean plasma concentrations of SUL-138 were used to determine mean toxicokinetic parameters for the rat. In addition, minimum and maximum plasma concentrations were used to determine toxicokinetic parameters and provide an indication for variability. For the minipig toxicokinetic parameters were determined per individual.

### 2.7 Statistical analysis

Data are presented as mean ± Standard Deviation (SD) except in the section describing rat toxicokinetics where data are presented as mean (minimum – maximum). R version 4.2.3 was used to perform an ANOVA followed Tukey’s HSD post hoc analysis to compare group means of continuous data [25]. In addition, categorical data were analyzed by Chi-square test with Yates’ continuity correction. Differences with p values < 0.05 were considered to be significant.

### 2.8 Ethical approval

The test facility and procedural aspects of both the rat and the minipig study were in compliance with Directive 2010/63/EU on the protection of animals used for scientific purposes. Studies were approved by the local animal-welfare body and the facility was authorized by the Italian ministry to use laboratory animals (No. 363/2021-PR). Studies were conducted for regulatory purposes and in compliance with relevant OECD and International Council for Harmonisation (ICH) guidelines.

## 3 Results

### 3.1 General toxicity rat

SUL-138 was well tolerated by male and female rats when orally administered up to 136 mg/kg for 30 days in this GLP repeated-dose general toxicity study. Clinical observations showed a slight to marked staining of the tongue on the right-side of the mouth indicative for local irritation potentially caused by the low pH of the dose formulations (pH= 4.73 for 27 mg/kg and pH= 2.75 for 682 mg/kg). It was observed in 2/10 (M and F, p= 0.07) from the 27 mg/kg group, 10/10 (M, p< 0.01) and 6/10 (F, p< 0.01) in the 136 mg/kg group and 6/15 (M, p< 0.05) and 11/15 (F, p< 0.01) in the 682 mg/kg dose group. The onset of this clinical sign was from approximately treatment day 17 and endured up to necropsy or recovery day 7 or 10 for recovery males and females dosed with 682 mg/kg, respectively. Dark staining incidence increased dose-dependently and was completely reversed by the end of the recovery period.

There were three unscheduled mortalities in the groups treated with 682 mg/kg SUL-138; one main male and female on dosing day 17 and one female satellite on day 20 (p= 0.14). Mortality had occurred overnight and the rats were discovered in the early morning. Clinical observations in these animals showed discoloration of the tongue and mouth. Complete necropsy of the main animals revealed a red to brown staining around the muzzle and missing of the tip of the tongue with microscopic findings of ulcers. Autolysis, graded mild to marked, hampered the microscopic evaluation of other macroscopic findings such as a white foamy content in the lung, dark depressed areas in the stomach and discoloration of the mesenteric lymph nodes. Findings of tubular basophilia in the kidney and hepatocellular vacuolation in the liver were consistent with microscopic findings in the 682 mg/kg dose group euthanized after 30 days of treatment. Consequently, the cause of death could not be established.

**Fig. 1:**
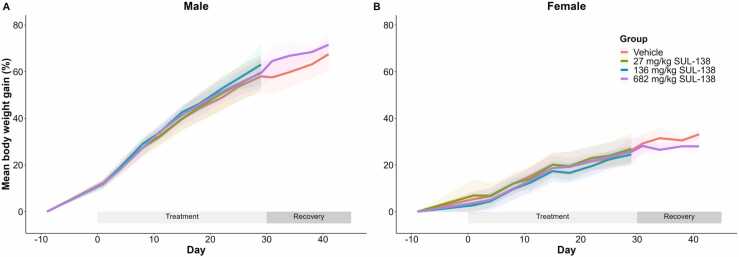
Mean body weight gain of male (A) and female (B) Sprague Dawley rats following oral treatment with SUL-138 for 1 month. After 1 month of treatment both the vehicle and high dose groups entered a recovery period for 14 days. Treatment with SUL-138 up to 682 mg/kg had no effect on body weight gain in male and female rats during the treatment and recovery phase. Shaded area represents the standard deviation (SD), n= 9–15 during the treatment phase and n= 5 during the recovery phase.

Further, there were no treatment-related effects on mean body weight gain (Fig. 1), food intake, body temperature and ophthalmoscopic observations for dose levels up to 682 mg/kg in both sexes.

Clinical pathology parameters revealed no abnormal changes in coagulation and urinalysis for dose levels up to 682 mg/kg. Hematology studies showed increased reticulocytes at the end of treatment in females dosed at ≥ 136 mg/kg (p< 0.05). Further, in females, eosinophils were decreased (29%, p< 0.05) and monocytes (60%, p< 0.05) were increased in the 136 and 682 mg/kg dose groups respectively. All changes in hematology parameters were absent at the end of the recovery period indicating complete reversibility. In the absence of other related changes, these changes in hematology were considered to be not adverse. At the end of the treatment period dose-dependent increases in plasma cholesterol levels were observed from dose levels ≥ 136 mg/kg for males and 682 mg/kg for females (Table 3). Additionally, increased plasma Alkaline Phosphatase (ALP) levels were noted in males treated with 136 mg/kg (22%, p< 0.01). In females, increased plasma protein levels (Protein, 6.4%, p< 0.01; Albumin, 5.0%, p< 0.01; Globulin, 8.9%, p< 0.05) and calcium levels (7.8%, p< 0.01) were measured in the 682 mg/kg dose group. A decrease in plasma chloride levels was also observed in females treated with 682 mg/kg (3%, p< 0.01). Observed statistical significant changes in clinical chemistry parameters are relative small biological variations of minor relevance and all were normalized at the end of recovery indicating full reversibility. However, for females, chloride levels were still lowered after recovery but the difference was minimal, only 2% compared to vehicle (p< 0.05). In males treated with 682 mg/kg, increased protein (4.3%, p< 0.05), globulin (8.6%, p< 0.05) and decreased A/G ratio (6.1%, p< 0.05) were recorded at the end of recovery which were not observed after the treatment phase. These small changes were therefore considered incidental and not related to treatment.

**Table 3:** Clinical pathology, urinalysis and histopathology of the rat after repeated administration of SUL-138 and after recovery.

**Sex**	**Male**						**Female**					
**Group**	**Main**				**Recovery**		**Main**				**Recovery**	
**Dose (mg/kg)**	**0**	**27**	**136**	**682**	**0**	**682**	**0**	**27**	**136**	**682**	**0**	**682**
Mean terminal body weight (g)	345.13 ± 17.30	344.07 ± 21.31	348.24 ± 9.48	339.06 ± 18.53	367.90 ± 18.86	367.96 ± 19.52	223.51 ± 10.10	226.47 ± 11.92	218.65 ± 8.75	224.24 ± 13.64	238.04 ± 13.99	227.62 ± 5.58
**Kidneys**												
Urinary volume (mL)	4.10 ± 1.97	5.40 ± 1.37	4.30 ± 1.16	5.50 ± 2.46	3.40 ± 2.07	3.60 ± 1.39	2.45 ± 1.66	2.40 ± 1.04	2.45 ± 1.04	3.83 ± 1.85	1.80 ± 1.10	1.50 ± 0
Kidney weight (g)	2.522 ± 0.18	2.446 ± 0.15	2.515 ± 0.13	2.564 ± 0.19	2.469 ± 0.15	2.765* ± 0.21	1.506 ± 0.14	1.522 ± 0.09	1.456 ± 0.08	1.541 ± 0.11	1.465 ± 0.18	1.525 ± 0.09
Kidney/body weight (%)	0.73 ± 0.04	0.71 ± 0.03	0.72 ± 0.03	0.76 ± 0.05	0.67 ± 0.04	0.75* ± 0.07	0.67 ± 0.04	0.67 ± 0.04	0.67 ± 0.03	0.69 ± 0.05	0.61 ± 0.05	0.67 ± 0.04
Tubular basophilia (n)	10/10	8/10	7/10	9/9*	3/5	5/5	3/10	3/10	4/10	8/9*	2/5	4/5
Tubular dilation (n)	5/10	5/10	5/10	5/9*	3/5	4/5	0/10	2/10	3/10	6/9*	0/5	2/5
Casts (n)	0/10	0/10	4/10	6/9*	0/5	0/5	0/10	1/10	1/10	3/9	0/5	0/5
Hyaline droplets (n)	0/10	0/10	2/10	6/9*	0/5	0/5	0/10	0/10	0/10	0/9	0/5	0/5
**Liver**												
Plasma cholesterol (mmol/L)	1.85 ± 0.20	1.92 ± 0.16	2.22**± 0.08	2.77***± 0.37	1.61 ± 0.18	1.70 ± 0.24	2.46 ± 0.34	2.41 ± 0.32	2.58 ± 0.46	3.59***± 0.57	2.21 ± 0.57	2.30 ± 0.58
Liver weight (g)	10.173 ± 0.82	10.285 ± 0.76	11.196* ± 0.29	13.217*** ± 1.06	9.741 ± 0.54	11.746*± 0.94	6.357 ± 0.66	6.390 ± 0.67	6.402 ± 0.40	7.820*** ± 1.09	6.138 ± 0.88	6.290 ± 0.45
Liver/body weight (%)	2.95 ± 0.17	2.99 ± 0.10	3.22**± 0.10	3.89*** ± 0.24	2.65 ± 0.16	3.21* ± 0.33	2.84 ± 0.22	2.82 ± 0.20	2.93 ± 0.16	3.48*** ± 0.39	2.57 ± 0.24	2.77 ± 0.24
Hepatocellular vacuolation (n)	0/10	0/10	0/10	6/9*	0/5	1/5	0/10	0/10	0/10	2/9	0/5	0/5
Hepatocyte hypertrophy (n)	0/10	0/10	0/10	2/9	0/5	0/5	0/10	0/10	0/10	0/9	0/5	0/5

(* p-value < 0.05 compared to vehicle; ** p-value < 0.05 compared to vehicle and 27 mg/kg; *** p-value < 0.05 compared to vehicle, 27 and 136 mg/kg)

**Fig. 2:**
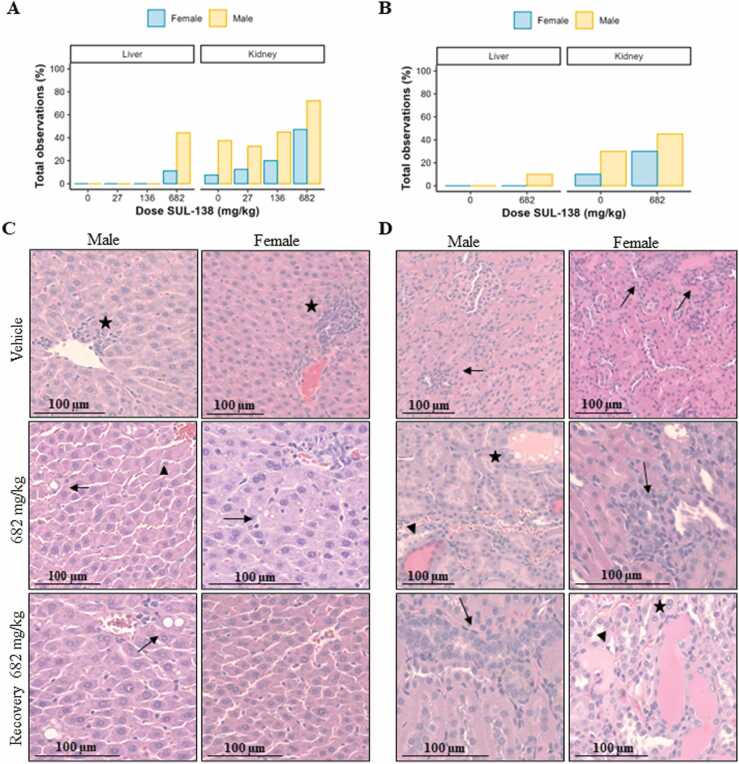
Microscopic observations in liver and kidney in male and female rats. Total microscopic observations in main groups after 30 days of treatment (A). Total microscopic observations in recovery groups after 30 days of treatment followed by 14 days recovery (B). Representative HE stainings of the liver (C). Stars indicate inflammatory cell foci (Vehicle), arrows indicate hepatocellular vacuolation and triangle indicates hypertrophy (Treatment groups). Representative HE stainings of the kidney (D). Arrows indicate tubular basophilia, triangles indicate tubular dilation and stars indicate casts.

Necropsies were performed to assess macroscopic and microscopic pathology. There were no differences in terminal body weight when comparing the vehicle with the treatment groups. Macroscopic inspection revealed a pale and or swollen liver in 3/9 males treated with 682 mg/kg (p< 0.05). In females, no macroscopic abnormalities were detected in the liver. Increases in absolute and relative liver weight were present in males treated with ≥ 136 mg/kg and in females dosed with 682 mg/kg (Table 3). Microscopic inspection of the liver revealed midzonal to panlobular hepatocellular vacuolation, graded minimal to mild, in 6/9 males and 2/9 females dosed with 682 mg/kg, p< 0.05 and p= 0.41 respectively (Fig. 2). Vacuoles were small and sharply demarcated and interpreted as fat accumulation. In addition, hepatocellular hypertrophy was observed in 2/9 males treated with 682 mg/kg (p< 0.05). At the end of the recovery period severity/incidence of the liver weight and microscopic observations indicated almost complete recovery in males and full recovery in females. In summary, increases in plasma cholesterol levels were accompanied with macroscopic findings in the liver, increased liver weights and microscopic findings.

In addition, at the end of the treatment period there were microscopic findings in the kidney from dose levels ≥ 136 mg/kg for males and 682 mg/kg for females. Tubular basophilia, tubular dilation and casts were present in male and female animals (Table 3**,** Fig. 2). In addition, hyaline droplet accumulation in tubular epithelial cells was only seen in males at dose levels ≥ 136 mg/kg. At the end of the recovery period, tubular basophilia was still present in both males and females of the 682 mg/kg dose group, although reduced in severity, when compared to vehicle, indicating partial recovery. There was complete recovery for all other microscopic findings in the kidney.

Organ weight, macroscopic and microscopic findings in all other tissues of SUL-138 groups were occurring with a similar incidence when comparing vehicle with treatment groups or were within the range of spontaneous findings. Therefore, these findings were considered to be not related to treatment.

### 3.2 Toxicokinetics of SUL-138 in the rat are dose-proportional without accumulation

**Fig. 3:**
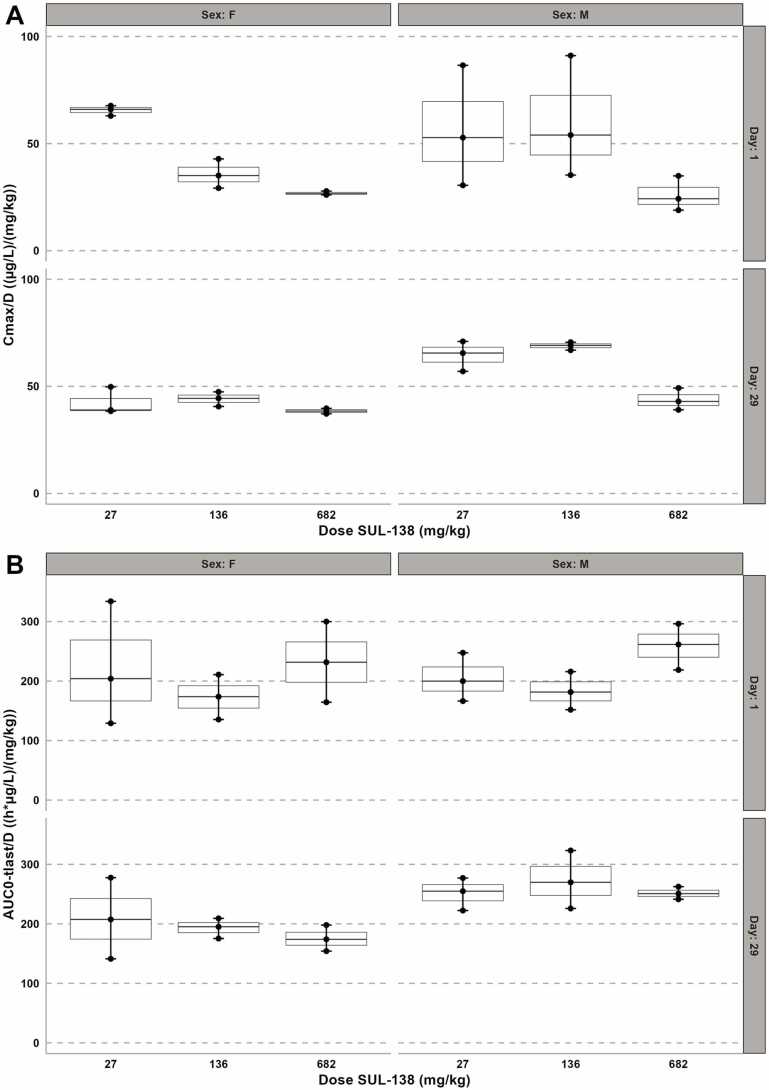
Mean, min and max dose-normalized toxicokinetic parameters C_max_ and AUC_0-tlast_ in male and female rats following oral administration of SUL-138 on day 1 and 29. AUC_0-tlast_ is dose-proportional in both males and females. Data is presented as mean (line) with minimum and maximum whiskers including datapoints (n= 3).

After a single and repeated-dose oral dose SUL-138 was rapidly absorbed with a T_max_ ranging between 0.13 – 0.5 (males) and 0.13 – 1.0 (females) hours at all dose levels with peak plasma concentration (C_max_) ranging between 1.44–29.3 ×10^3^µg/L in males, and 1.06–26.2 ×10^3^µg/L in females (Table S1). SUL-138 was eliminated from the systemic circulation with a t_1/2_ ranging between 3.9–7.9 hours for males and 4.5–7.2 hours for females at all dose levels. At approximately 4.0 hours post-dose a slight increase in drug plasma concentration was observed suggestive of enterohepatic recirculation (Figure S1). The systemic dose-corrected exposure (AUC_0-tlast_/D), did not differ between dose groups taking into account the observed variation indicated by min and max values (Fig. 3/Table S2). Exposure (AUC_0-tlast_) increased in a dose proportional manner. Overall, total exposure on day 1 and day 29 was similar between dosing groups: AUC_0-tlast_ ranged between 5.45–178 ×10^3^ h*µg/L in males and 5.56–158 ×10^3^ h*µg/L in females. The reduction in total exposure for both males and females treated with 682 mg/kg at day 29 suggests increased (first-pass) metabolism or excretion at higher dosing. In addition, SUL-138 was detected in predose plasma concentrations on day 29 in the high dose group at higher concentrations compared to the 24 hours sample, however this difference is not reflected in the total exposure. Further, the male:female exposure in terms of AUC_0-∞_, AUC_0-tlast_ and C_max_ ranged from 0.91 to 1.18 at day 1 and from 1.12 to 1.68 on day 29, indicating a trend towards higher exposure in males at day 29 (Table S2).

### 3.3 General toxicity minipig

SUL-138 was well tolerated by male and female minipigs when orally administered up to 409 mg/kg for 30 days in this GLP repeated-dose general toxicity study. There was no mortality. Clinical signs, as indicator for potential toxicity of SUL-138, did not reveal any treatment-related effects for dose levels up to 82 mg/kg. Incidentally, two males receiving 16 mg/kg showed a left inguinal swelling from treatment day 22 to day 31 or emesis at day 29 (p= 0.78). However, these clinical signs were considered sporadic and not related to SUL-138 treatment. Further, during the first four days of the study, treatment-related clinical signs were observed after administration of 409 mg/kg in 3/5 males (p= 0.16) and 4/5 females (p= 0.052). The main clinical signs were decreased activity, emesis and tremors which were observed 2.5 – 3.5 hours post-dose in the first dosing days.

SUL-138 plasma concentrations measured on day 1 at 2 and 4 hours post-dose ranged between 1.06 ×10^4^ - 2.62 ×10^3^ and 1.58 ×10^3^ – 1.93 ×10^3^ µg/L for males and females respectively. Additionally, involuntary contraction of muscles was observed in 1/5 males treated with 409 mg/kg on treatment day 1 at 2.0 – 2.5 hours post-dose and interpreted as clinical signs of convulsions (p= 0.29). The mean SUL-138 plasma concentration determined at 2 hours was 1.06 ± 0.17 ×10^4^ µg/L which is close to the mean C_max_ of 1.06 ± 0.16 ×10^4^ µg. In addition, the onset of convulsions was close to the T_max_ of 1.7 ± 0.6 hours. For females, peak plasma concentrations on day 1 were determined at a C_max_ of 6.36 ± 5.0 ×10^3^ µg/L with T_max_ of 1.6 ± 2.1 hours (Table S3). All minipigs fully recovered after experiencing clinical signs and no clinical signs were recorded from treatment day 5 onwards except for one sporadic episode. Decreased activity and involuntary contraction of muscles interpreted as convulsions were recorded on day 16 and 18 in 1/5 females treated with 409 mg/kg (p= 0.29).

**Fig. 4:**
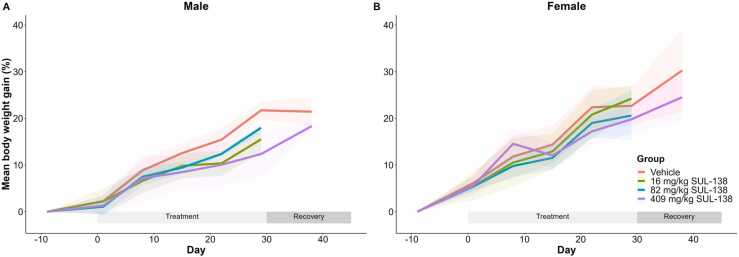
Mean body weight of male (A) and female (B) Göttingen minipigs following oral treatment with SUL-138 for 1 month. After 1 month of treatment both the vehicle and high dose groups entered a recovery period for 14 days. Treatment with SUL-138 up to 409 mg/kg had no effect on body weight gain in male and female minipigs during the treatment and recovery phase. Shaded area represents the standard deviation (SD), n = 3–5 during the treatment phase and n = 2 during the recovery phase.

Other clinical observations that were performed during the treatment and recovery phase showed no adverse effects of SUL-138. There were no treatment-related effects on mean body weight gain (Fig. 4), food intake, body temperature, electrocardiographic tracings and ophthalmoscopic observations.

Next, clinical pathology parameters were evaluated and revealed no abnormal changes in hematology and urinalysis for dose levels up to 409 mg/kg in males and females. For coagulation, an increased mean prothrombin time was recorded at the end of treatment in females dosed at 409 mg/kg compared to vehicle (17%, p< 0.05), however this increase was not observed at the end of the recovery period. Due to the absence of other related changes and minimal severity it was considered to be not adverse. Similarly, in the clinical chemistry study, lowered triglyceride levels were recorded at the end of treatment (46%, p< 0.05) and recovery (46%, p= 0.11) in males dosed at 409 mg/kg compared to vehicle. Triglyceride levels were still within the normal range of historical data. In addition, plasma urea levels (43%, p< 0.05) were lowered in males dosed at 16 mg/kg and cholesterol levels (39%, p< 0.05) were lowered in females dosed at 82 mg/kg. However, in comparison with the vehicle mean recorded prior to treatment urea (29%, p< 0.05) and cholesterol (23%, p= 0.32) levels were already lowered in these groups. Recorded changes in clinical chemistry parameters were therefore considered incidental and not related to treatment.

Necropsies were performed on all animals from the main group. All findings of macroscopic pathology and organ weights were within the range of expected spontaneous findings. Additionally, all tissues appeared normal at microscopic evaluation for dose levels up to 409 mg/kg in both sexes.

### 3.4 Toxicokinetics minipig

**Fig. 5:**
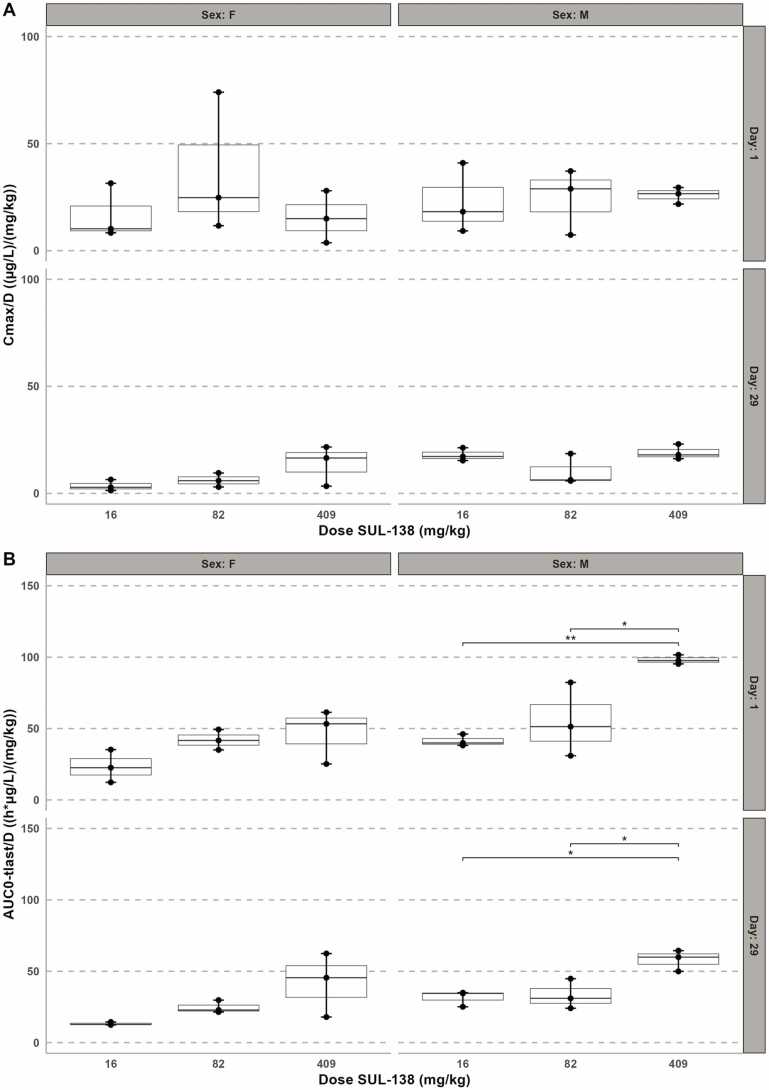
Dose-normalized toxicokinetic parameters C_max_ (A) and AUC_0-tlast_ (B) in male and female minipigs following oral administration of SUL-138 on day 1 and 29. C_max_ is dose proportional in both males and females (p > 0.05). AUC_0-tlast_/D increased with increasing dose levels. (*p< 0.05, **p< 0.01, n= 3).

After single and repeated-dose oral doses SUL-138 was rapidly absorbed with a mean T_max_ ranging between 0.17 ± 0.07 – 1.67 ± 0.58 and 0.54 ± 0.44 – 1.58 ± 2.10 hours at all dose levels for males and females respectively. The dose-corrected maximum plasma concentration, C_max_/D, shows no difference over the dose range which indicates dose-proportionality for both sexes (Fig. 5). Elimination from the systemic circulation had a t_1/2_ ranging between 3.6 ± 0.7 – 8.6 ± 0.2 hours for males and 1.4 ± 0.6 – 4.7 ± 1.0 for females (Table S3). At approximately 4.0 hours post-dose a slight increase in drug plasma concentration was observed in females dosed with 409 mg/kg, suggestive of enterohepatic recirculation (Figure S2). Further, the systemic exposure (AUC_0-tlast_) increased with the dose levels: values ranged between 677 ± 68 and 40.1 ± 1.3 ×10^3^ h*µg/L on day 1, and between 516 ± 91 and 23.7 ± 3.0 ×10^3^ h*µg/L on day 29 in males. Similar, in females AUC_0-tlast_ ranged between 382 ± 188 and 19.1 ± 7.8 ×10^3^ h*µg/L and 216 ± 18 and 17.1 ± 9.2 ×10^3^ h*µg/L on treatment day 1 and 29 respectively. The reduction in C_max_ and AUC_0-tlast_ for both males and females at day 29 of SUL-138 and suggests an increased (first-pass) metabolism or excretion.

In addition, predose SUL-138 plasma concentrations on day 29 in all dose groups were below LLOQ or very close to LLOQ (19.46 µg/L, males, 409 mg/kg) which also denote absence of accumulation. Further, male:female exposure in terms of AUC_0-∞_, AUC_0-tlast_ and C_max_ ranged from 0.66 to 2.11 at day 1 and from 0.61 – 5.05 on day 29, indicating a trend towards higher exposure in males (Table S4).

## 4 Discussion

The present study was designed to support a FIH clinical trial and characterize the toxicological, safety and toxicokinetic profile of SUL-138. Therefore, SUL-138 was investigated in 30-day GLP repeated dose toxicity studies in rats and minipigs following EMA and FDA guidelines. Vehicle treatment and three dose levels of SUL-138, spaced by a fivefold interval, were investigated to establish a NOAEL. In the rat study it was shown that repeated oral administrations of SUL-138 for 30 consecutive days were well tolerated up to dose levels of 136 mg/kg without adverse findings. In the minipig study, dose levels up to 82 mg/kg were well tolerated without any adverse findings. Even though minipigs receiving 409 mg/kg experienced transient clinical signs during the first treatment days, there were no adverse findings after stopping the treatment. Given these outcomes in the rat and minipig study, respective 30-day oral NOAELs were considered to be 136 and 82 mg/kg.

In the rat study, three unscheduled mortalities were observed overnight after 17 days of repeated 682 mg/kg SUL-138 administration. Clinical observations in these rats indicated local irritation in the mouth potentially caused by acidity of the 682 mg/kg dose formulation (pH= 2.75). According to literature recommendations for the pH of oral dose formulations range from 2 – 9 [27] or 4.5/5 – 9 [28], [29]. Despite that there is no consensus regarding the lower pH limit the high dose SUL-138 formulation is within the recommended range and likely near this lower limit. Further, macroscopic observations revealed a white foamy content in the lungs which could be indicative for a technical gavage error or gavage-related reflux. Both can result from aspiration of the formulation or irritant stomach contents. Microscopic interpretation of tissues was impeded by autolysis but findings of tubular basophilia and hepatocellular vacuolation were consistent with findings in the 682 mg/kg dose group euthanized after 30 days of treatment. Hence, the cause of death was not established. Collectively, these data suggest a local rather than a systemic effect as a result of the low pH of the oral dose formulation possibly in combination with a technical error contributing to the death. However, a relationship with high SUL-138 dosing cannot be completely excluded.

The results of the GLP toxicity study in rats also showed at the end of the treatment period dose-dependent increases in plasma cholesterol levels from dose levels ≥ 136 mg/kg for males and 682 mg/kg for females. These changes in plasma cholesterol levels match with macroscopic observations of the liver, increases in absolute and relative liver weight and microscopic findings of hypertrophy and vacuolation. Importantly, there was near complete recovery in males and complete recovery in females two weeks following cessation of treatment. Liver enlargement is associated with microsomal CYP450 enzyme induction which is commonly accompanied by findings of hepatocellular hypertrophy [30]. The increase in plasma cholesterol and hepatic vacuolation, interpreted as fat accumulation, could suggest Peroxisome Proliferator-Activated Receptor α (PPARα) mediated microsomal enzyme induction [31]. In rats PPARα is highly expressed in the liver, whereas expression and responsiveness in other species such as humans is considerably lower [32], [33]. Further, toxicokinetic analysis showed a decrease in systemic exposure to SUL-138 in rats dosed with 682 mg/kg. Taken together, these findings and their reversibility suggest an adaptive non-adverse response to enzyme induction following high SUL-138 exposure [31]. Besides the liver, tubular basophilia, dilation, casts and hyaline droplets were observed in the kidney. The incidence of tubular basophilia was similar between control and SUL-138 treatment groups in both sexes. In addition, incidence of tubular dilation was similar between control and treatment groups in males. SD rats, used in the present study, are especially vulnerable to develop spontaneous renal lesions wherein tubular basophilia precedes tubular dilation and cast formation [34]. Overall, these findings indicate the development of renal pathologies unrelated to SUL-138 treatment and it is commonly referred to as Chronic Progressive Nephropathy (CPN). Further, the hyaline droplets were only observed in male rats and fully reversible after recovery. Hyaline droplets are a hallmark for a sex and species-specific interference of lysosomal degradation of α2 u-globulin in rats. This protein is highly expressed in male rats, not in female rats or humans, and can bind xenobiotics or its metabolites which can interfere lysosomal hydrolysis leading to depositions of hyaline droplets [35], [36]. All other findings in the rat study were considered to be non-adverse and unrelated to SUL-138 treatment. Interestingly, in the minipig study, despite the transient clinical signs in the high dose group the first treatment days, there were no treatment-related adverse findings.

Further, our results demonstrate that toxicokinetic parameters C_max_ and AUC_0-tlast_ in the rat are dose-proportional in line with previous work [18]. In the minipig the C_max_ is dose-proportional, however AUC_0-tlast_ increased with dose levels which could be indicative for saturation of metabolism [37]. Importantly, when comparing predose plasma concentrations or systemic exposure levels in terms of AUC_0-tlast_ ratios on day 29 our data show that there is no accumulation of SUL-138 in both species. On the contrary, on day 29 there appears to be an increased metabolism or clearance in high dose rats and across all dose levels in the minipig. In rats this is in line with the dose-dependent increase in liver weight and is commonly referred to as an adaptive response after xenobiotic exposure [31]. Further, rat toxicokinetic analysis showed a tendency in sex differences in total exposure, however firm conclusions cannot be drawn given the limited sample size and absence of a full toxicokinetic profile per animal. Sex differences in exposure were more prominently seen in minipigs, e.g. > 1.3 fold higher exposure in females compared to males at low and high dose. Interestingly, a slight increase in SUL-138 plasma concentration was observed approximately 4 hours post-dose in high dose females suggesting enterohepatic recirculation. This may be responsible for increased exposure to SUL-138 and prolonging its half-life. Enterohepatic recirculation can be the result of glucuronidation by uridine 5′-diphosphate (UDP)-glucuronosyltransferases (UGTs) [38], [39], phase II metabolic pathway, which was also observed for SUL-138 in minipig and human hepatocyte metabolism studies [18]. Given the presented toxicokinetic results, future *in vivo* Absorption Distribution Metabolism and Excretion (ADME) studies with radiolabeled SUL-138 may further characterize the compound’s metabolism and metabolites. In perspective, for future clinical trials with patients the metabolic profile of SUL-138 must be elucidated to definitely exclude significant exposure to metabolites in humans.

One limitation of the current study is that emesis could have affected the toxicokinetic analysis in minipigs. Emesis was recorded in two females treated with 409 mg/kg on day 1 and one male treated with 16 mg/kg on day 29. Both females and the male had C_max_ and AUC_0-tlast_ values in the upper range of the mean group outcome. On the contrary, emesis occurred approximately 2 hours after C_max_/T_max_ which suggests that most of the administered SUL-138 had already been absorbed thereby reducing the overall impact on toxicokinetic analysis. Further, in the females dosed with 409 mg/kg, a higher degree of variation in toxicokinetic parameters was observed possibly contributing to differences in systemic exposure. However, gastric emptying could also play a role and there is currently no scientific consensus regarding gastric emptying time for the Göttingen minipig. These have been to found to be subject to a high degree of variability and are reported in the range of 0.63 hours for liquids after overnight fasting [40] or 2.3 – 8.4 hours depending on nutritional status [41]. From an experimental and toxicological point of view, inducing clinical signs such as emesis was unavoidable. Regulatory bodies and expert panels of toxicologists recommend that the high dose level should induce non-debilitating toxicity which is essential for an adequate hazard identification of a new chemical entity [22], [23], [42], [43]. In addition, substitution of the first SUL-138 plasma concentration below LLOQ value by 0.5 * LLOQ, for calculation purposes, could result in a slight overestimation of the AUC_0-tlast_ and half-life.

In summary, SUL-138 is a promising orally bioavailable therapeutic for treatment of NCDs through improvement of mitochondrial function [18], [19], [20]. Regulatory compulsory toxicology studies demonstrated that SUL-138 is well tolerated in rat and minipig. There were no safety concerns raised for common target organs following repeated oral administration. Further, toxicokinetic evaluation showed in general that SUL-138 is rapidly absorbed, eliminated from the systemic circulation and does not accumulate. The next step for the preclinical to clinical transition of SUL-138 is to perform a thorough *in vivo* preclinical ADME characterization and subsequently a FIH clinical trial to asses safety and tolerability in humans.

## CRediT authorship contribution statement

**Daniël Henri Swart:** Writing – original draft, Visualization, Formal analysis, Data curation. **Guido Krenning:** Writing – review & editing, Writing – original draft, Visualization, Formal analysis. **Daan J. Touw:** Writing – review & editing, Writing – original draft, Visualization, Formal analysis. **Nadir Ulu:** Writing – review & editing. **Adrianus C. van der Graaf:** Data curation. **Sovan Adel:** Data curation. **Rob H. Henning:** Writing – review & editing. **Jasper Stevens:** Writing – review & editing. **Martin de Haan:** Writing – review & editing, Formal analysis, Data curation.

## Declaration of Competing Interest

The authors declare the following financial interests/personal relationships which may be considered as potential competing interests: D.H. Swart is Chief of Operations, M. de Haan is senior consulting toxicologist, S. Adel is research technician, A.C. van der Graaf is Chief Executive Officer, G. Krenning is Chief Scientific Officer at Sulfateq B.V. (Groningen, The Netherlands) a company that owns patents on SUL-138 and funded the presented study. R.H. Henning chairs the Scientific Advisory Board of Sulfateq B.V. N. Ulu is Vice President R&D and Clinical Operations at Gen İlaç ve Sağlık Ürünleri A.Ş. (Ankara, Turkey) a company which is license-holder for the use of SUL-138 as a therapeutic for neurodegenerative disease.

## Data Availability

Data will be made available on request.
