# Supplementary material for: Safety, tolerability and toxicokinetics of the novel mitochondrial drug SUL-138 administered orally to rat and minipig
Source: Toxicol Rep. 2024 Mar 19;12:345–55. doi: 10.1016/j.toxrep.2024.03.009 (PMC10981007; doi:10.1016/j.toxrep.2024.03.009)
Supplement: Supplementary file 1 — Supplementary material. [file mmc1.pdf]

Supplementary Data

Table S1: Mean, min and max toxicokinetic parameters for SUL-138 in plasma from male and female Sprague Dawley rats following repeated oral administration once daily in a 30-day GLP toxicity study (n= 3).

		Day 1				Day 29					
**Sex**	**Dose (mg/kg)**	**C_{max} (µg/L)**	**T_{max} (h)**	**$AUC_{0-tlast}$ (h*µg/L)**	**$t_{1/2}$ (h)**	**C_{max} (µg/L)**	**T_{max} (h)**	**$AUC_{0-tlast}$ (h*µg/L)**	**$t_{1/2}$ (h)**	**$C_{max}R$ Day 29/Day 1**	**AUCR Day 29/Day 1**
M	27	1439 (832 - 2359)	0.13 (0.13 - 0.13)	5450 (4536 - 6748)	4.6 (4.6 - 4.9)	1787 (1554 - 1935)	0.25 (0.25 - 0.25)	6948 (6062 - 7549)	4.0 (4.0 - 4.1)	1.24	1.27
	136	7358 (4813 - 12412)	0.13 (0.13 - 0.5)	24740 (20690 - 29420)	4.7 (4.5 - 4.7)	9420 (9122 - 9623)	0.5 (0.5 - 0.5)	36775 (30778 - 44056)	3.9 (3.9 - 4)	1.28	1.49
	682	16525 (12896 - 23802)	0.5 (0.25 - 0.5)	178196 (149035 - 201898)	7.9 (7.0 - 8.0)	29287 (26611 - 33533)	0.25 (0.25 - 0.25)	170858 (164455 - 178860)	6.5 (6.4 - 6.5)	1.77	0.96
F	27	1799 (1716 - 1845)	0.5 (0.5 - 0.5)	5563 (3519 - 9104)	5.0 (4.8 - 5.5)	1062 (1047 - 1357)	0.25 (0.13 - 0.25)	5656 (3852 - 7565)	4.5 (3.2 - 5.2)	0.59	1.02
	136	4781 (3982 - 5840)	1.0 (0.25 - 1.0)	23678 (18458 - 28733)	4.7 (4.5 - 4.8)	6053 (5535 - 6464)	0.25 (0.25 - 0.25)	26591 (23897 - 28510)	6.0 (5.3 - 6.5)	1.27	1.12
	682	18124 (17763 - 18963)	0.25 (0.25 - 0.5)	157871 (112074 - 204380)	7.1 (6.3 - 7.7)	26213 (25354 - 27072)	0.13 (0.13 - 0.13)	118576 (105065 - 134878)	7.2 (5.6 - 9.8)	1.45	0.75

Abbreviations: C_{max}: maximum plasma concentration. T_{max}: time of maximum plasma concentration. $AUC_{0-tlast}$: area under the curve from t = 0 to t_{last}. $t_{1/2}$: terminal half-life. $C_{max}R$: ratio of C_{max} on day 29 divided by day 1. AUCR: ratio of $AUC_{0-tlast}$ on day 29 divided by day 1.

Table S2: Mean, min and max dose-normalized toxicokinetic parameters and male:female sex ratio for SUL-138 in plasma from male and female Sprague Dawley rats following repeated oral administration once daily in a 30-day GLP toxicity study (n= 3).

		Dose-normalized toxicokinetic parameters					
		Day 1			Day 29		
Parameter	Sex	27 mg/kg	136 mg/kg	682 mg/kg	27 mg/kg	136 mg/kg	682 mg/kg
$AUC_{0-\infty}$/Dose	M	204 (170 - 252)	185 (155 - 219)	297 (240 - 339)	258 (226 - 280)	273 (229 - 327)	268 (259 - 280)
	F	211 (134 - 345)	177 (138 - 215)	252 (181 - 323)	214 (216 - 291)	206 (182 - 223)	195 (164 - 248)
$AUC_{0-tlast}$/Dose	M	200 (166 - 248)	182 (152 - 216)	262 (219 - 296)	255 (222 - 277)	270 (226 - 323)	251 (241 - 262)
	F	204 (129 - 334)	174 (135 - 211)	232 (164 - 300)	208 (141 - 278)	195 (175 - 209)	174 (154 - 198)
C_{max}/Dose	M	53 (31 - 87)	54 (35 - 91)	24 (19 - 35)	66 (57 - 71)	69 (67 - 71)	43 (39 - 49)
	F	66 (63 - 68)	35 (29 - 43)	27 (26 - 28)	39 (38 - 50)	44 (41 - 47)	38 (37 - 40)

	Male:Female sex ratio					
	Day 1			Day 29		
Parameter	27 mg/kg	136 mg/kg	682 mg/kg	27 mg/kg	136 mg/kg	682 mg/kg
$AUC_{0-\infty}$	0.96	1.05	1.18	1.21	1.33	1.38
$AUC_{0-tlast}$	0.98	1.04	1.13	1.23	1.38	1.44
C_{max}	0.80	1.54	0.91	1.68	1.56	1.12

Abbreviations: C_{max}: maximum plasma concentration. $AUC_{0-tlast}$: area under the curve from t = 0 to t_{last}. $AUC_{0-\infty}$ area under the curve from t = 0 to infinity.

Table S3: Mean toxicokinetic parameters for SUL-138 in plasma from male and female Göttingen minipigs following repeated oral administration once daily in a 30-day GLP toxicity study (n= 2 - 3).

		Day 1				Day 29					
**Sex**	**Dose (mg/kg)**	**C_{max} (µg/L)**	**T_{max} (h)**	**$AUC_{0\text{-}tlast}$ (h*µg/L)**	**$t_{1/2}$ (h)**	**C_{max} (µg/L)**	**T_{max} (h)**	**$AUC_{0\text{-}tlast}$ (h*µg/L)**	**$t_{1/2}$ (h)**	**$C_{max}R$ Day 29/Day 1**	**AUCR Day 29/Day 1**
M	16	373 ± 268	0.92 ± 0.95	677 ± 68	8.6 ± 0.2	294 ± 50	0.17 ± 0.07	516 ± 91	5.6 ± 3.6	1.08 ± 0.7	0.76 ± 0.14
	82	2001 ± 1258	0.92 ± 0.95	4486 ± 2114	4.2 ± 0.4	836 ± 590	1.33 ± 0.58	2722 ± 861	5.2 ± 0.7	0.52 ± 0.33	0.67 ± 0.29
	409	10616 ± 1593	1.67 ± 0.58	40148 ± 1311	3.7 ± 0.7	7799 ± 1445	1.38 ± 1.08	23743 ± 3036	3.6 ± 0.7	0.73 ± 0.05	0.59 ± 0.09
F	16	273 ± 210	0.54 ± 0.44	382 ± 188	1.4 ± 0.6	58 ± 43	1.00 ± 0.87	216 ± 18	2.7 ± 0.3	0.33 ± 0.38	0.7 ± 0.42
	82	3011 ± 2687	0.75 ± 0.43	3436 ± 584	4.5 ± 0.9	504 ± 268	0.58 ± 0.38	2017 ± 365	4.7 ± 1.0	0.31 ± 0.24	0.59 ± 0.03
	409	6360 ± 4965	1.58 ± 2.10	19064 ± 7759	3.4 ± 0	5668 ± 3855	1.33 ± 0.58	17144 ± 9174	2.8 ± 0.1	0.93 ± 0.17	0.86 ± 0.15

Abbreviations: C_{max}: maximum plasma concentration. T_{max}: time of maximum plasma concentration. $AUC_{0\text{-}tast}$: area under the curve from t = 0 to t_{last}. $t_{1/2}$: terminal half-life. $C_{max}R$: ratio of C_{max} on day 29 divided by day 1. AUCR: ratio of $AUC_{0\text{-}tlast}$ on day 29 divided by day 1.

Table S4: Dose-normalized toxicokinetic parameters and male:female sex ratio for SUL-138 in plasma from male and female Göttingen minipigs following repeated oral administration once daily in a 30-day GLP toxicity study (n= 1 – 3).

		Dose-normalized toxicokinetic parameters					
		Day 1			Day 29		
**Parameter**	**Sex**	**16 mg/kg**	**82 mg/kg**	**409 mg/kg**	**16 mg/kg**	**82 mg/kg**	**409 mg/kg**
$AUC_{0-\infty}$/Dose	M	44 ± 3.4	68.5 ± 22	98.8 ± 3.0	33.7 ± 6.3	34.6 ± 11	58.5 ± 7.7
	F	34.1	43.5 ± 7.3	57.7 ± 5.7	55.3 ± 42.3	27.3 ± 5.3	54.5 ± 11.8
$AUC_{0-tlast}$/Dose	M	41.4 ± 4.2	54.9 ± 25.8	98.2 ± 3.2	31.5 ± 4.5	33.3 ± 10.5	58.1 ± 7.4
	F	23.4 ± 11.5	42.0 ± 7.1	46.6 ± 19.0	13.2 ± 0.9	24.7 ± 4.5	41.9 ± 22.4
C_{max}/Dose	M	22.8 ± 16.4	24.5 ± 15.4	26.0 ± 3.9	18.0 ± 2.5	10.2 ± 7.2	19.1 ± 3.5
	F	16.7 ± 12.9	36.8 ± 32.9	15.6 ± 12.1	3.6 ± 2.1	6.2 ± 3.3	13.9 ± 9.4

	Male:Female sex ratio					
	Day 1			Day 29		
**Parameter**	**16 mg/kg**	**82 mg/kg**	**409 mg/kg**	**16 mg/kg**	**82 mg/kg**	**409 mg/kg**
$AUC_{0-\infty}$	1.29	1.57	1.71	0.61	1.27	1.07
$AUC_{0-tlast}$	1.77	1.31	2.11	2.39	1.35	1.38
C_{max}	1.37	0.66	1.67	5.05	1.66	1.38

Abbreviations: C_{max}: maximum plasma concentration. $AUC_{0-tlast}$: area under the curve from t = 0 to t_{last}. $AUC_{0-\infty}$ area under the curve from t = 0 to infinity.

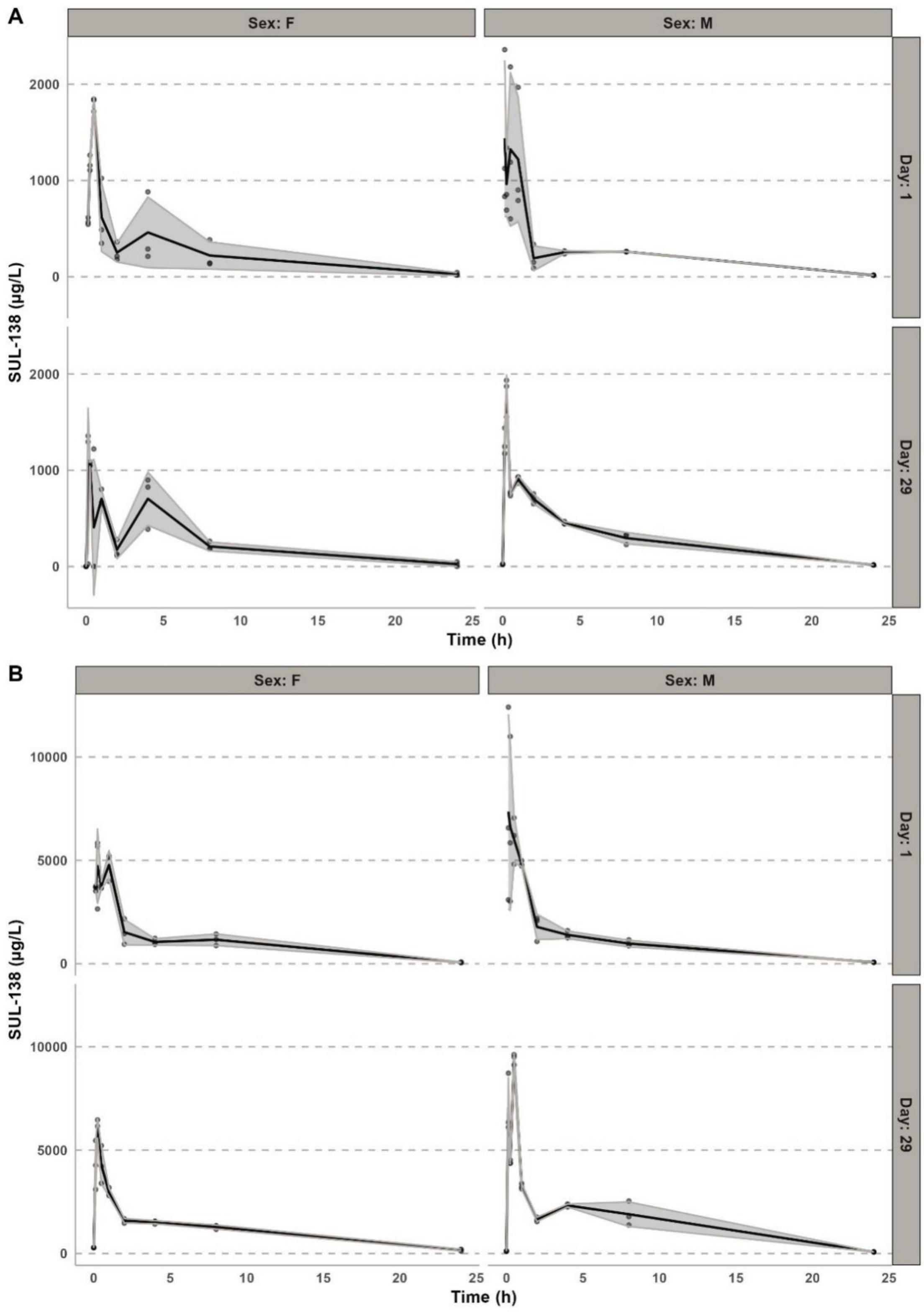

Figure S1: Toxicokinetic profile of SUL-138 in male and female Sprague Dawley rats. (A) 27 mg/kg and (B) 136 mg/kg.

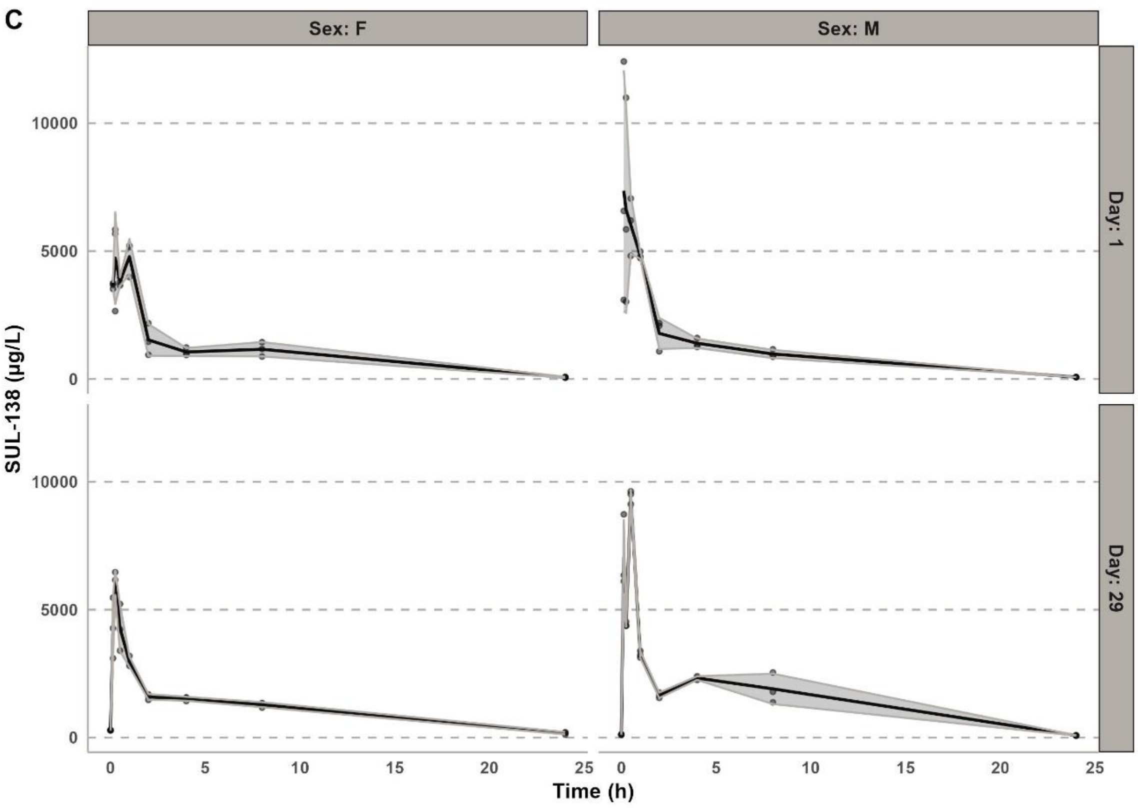

Figure S1: (Continued) Toxicokinetic profile of SUL-138 in male and female Sprague Dawley rats. (C) 682 mg/kg.

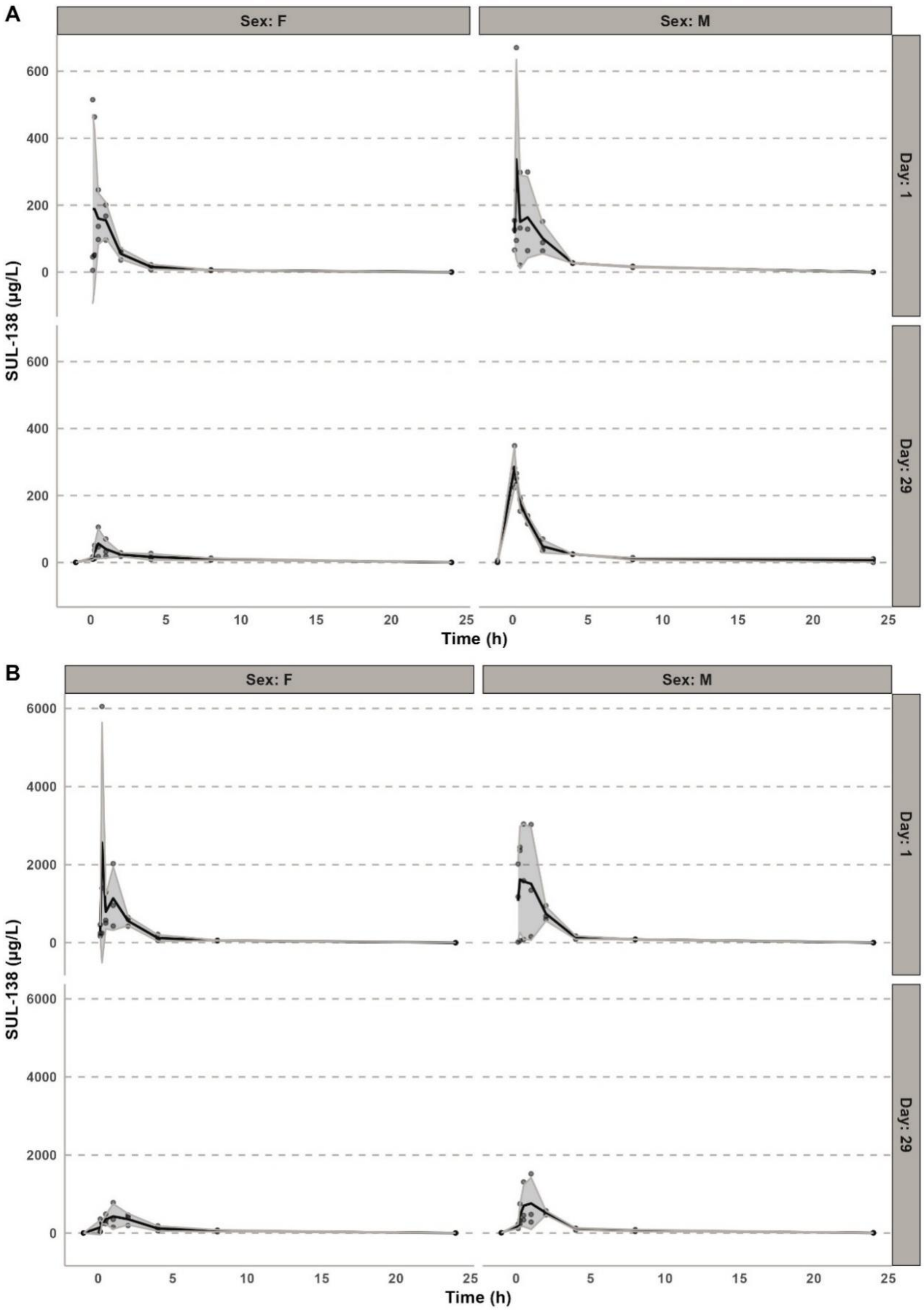

Figure S2: Toxicokinetic profile of SUL-138 in male and female Göttingen minipigs. (A) 16 mg/kg and (B) 82 mg/kg.

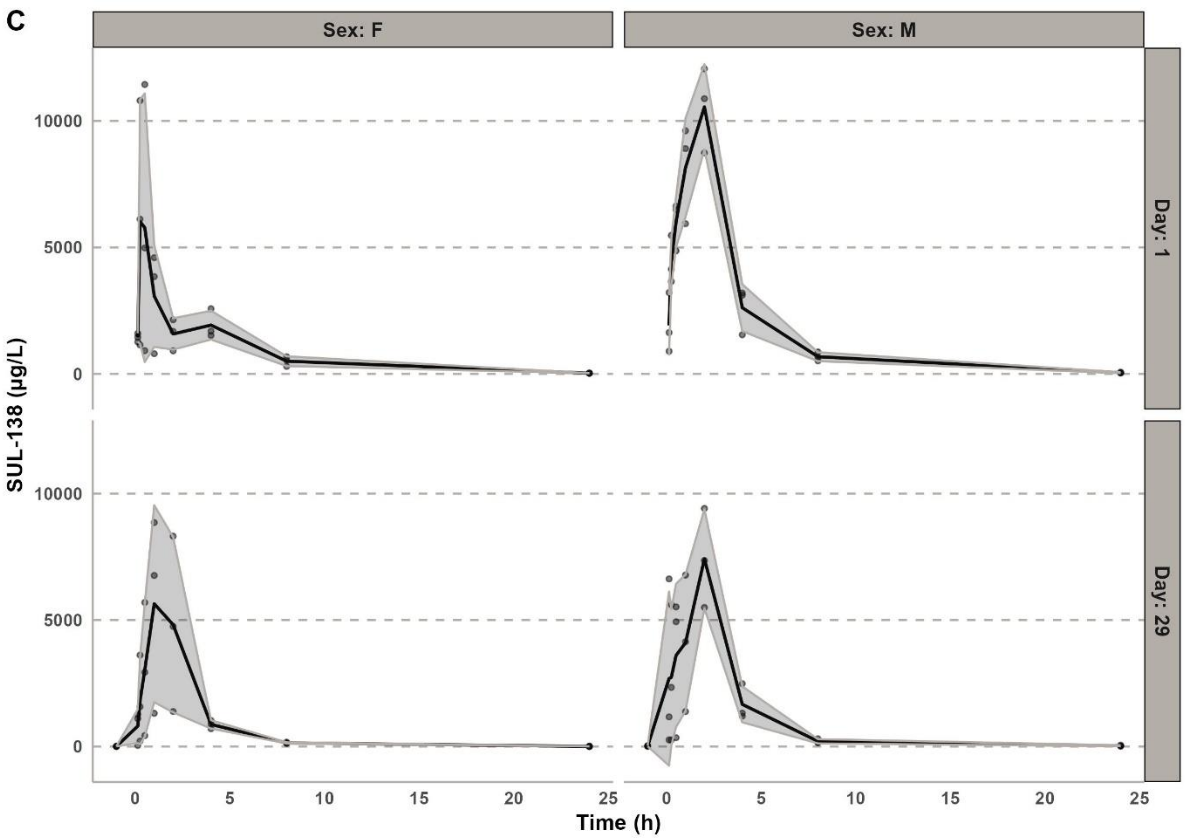

Figure S2: (Continued) Toxicokinetic profile of SUL-138 in male and female Göttingen minipigs. (C) 409 mg/kg.

Table S5: Mean clinical chemistry parameters in male and female Sprague Dawley rats at the end of treatment and recovery.

	Main				Recovery	
**Dose (mg/kg)**	**0**	**27**	**136**	**682**	**0**	**682**
**n**	**10**	**10**	**10**	**9**	**5**	**5**
**Males**						
ALP (U/L)	103 ± 10.8	105 ± 11.9	125* ± 13.2	106 ± 10.5	81.3 ± 10.1	78.7 ± 5.73
ALT (U/L)	40.7 ± 20.9	37.1 ± 13.4	36.7 ± 9.10	41.7 ± 7.95	34.2 ± 6.57	38.4 ± 13.0
AST (U/L)	125 ± 47.1	119 ± 23.7	111 ± 19.8	106 ± 15.6	101 ± 19.3	110 ± 14.8
TBIL (mg/dL)	0.05 ± 0.02	0.04 ± 0.01	0.04 ± 0.01	0.03 ± 0.02	0.05 ± 0.01	0.05 ± 0.01
CHOL (mg/dL)	71.7 ± 8.30	74.3 ± 5.99	86.0* ± 3.20	107* ± 14.1	62.3 ± 6.99	65.8 ± 9.13
GLU (mg/dL)	145 ± 23.3	148 ± 32.4	147 ± 30.9	141 ± 31.6	111 ± 15.3	117 ± 26.6
UREA (mg/dL)	50.3 ± 7.38	50.2 ± 5.43	45.7 ± 6.58	47.8 ± 5.13	46.9 ± 2.73	47.6 ± 3.55
CREA (mg/dL)	0.32 ± 0.04	0.35 ± 0.04	0.34 ± 0.04	0.33 ± 0.04	0.37 ± 0.02	0.38 ± 0.04
PROT (g/dL)	5.48 ± 0.24	5.48 ± 0.19	5.51 ± 0.14	5.57 ± 0.18	4.60 ± 0.17	4.80* ± 0.07
ALB (g/dL)	3.71 ± 0.14	3.71 ± 0.14	3.72 ± 0.11	3.70 ± 0.15	3.20 ± 0.12	3.28 ± 0.04
GLO (g/dL)	1.77 ± 0.13	1.77 ± 0.09	1.79 ± 0.06	1.87 ± 0.07	1.40 ± 0.07	1.52* ± 0.04
A/G	2.10 ± 0.12	2.12 ± 0.12	2.10 ± 0.08	1.99 ± 0.13	2.30 ± 0.12	2.16* ± 0.05
Cl (mmol/L)	101 ± 1.41	101 ± 0.93	100 ± 0.99	99.6 ± 0.86	97.7 ± 1.31	97.8 ± 1.44
Ca (mmol/L)	2.51 ± 0.10	2.52 ± 0.10	2.48 ± 0.08	2.60 ± 0.09	2.16 ± 0.05	2.23 ± 0.24
Na (mmol/L)	141 ± 1.39	142 ± 0.92	142 ± 0.89	142 ± 0.86	138 ± 0.72	137 ± 0.88
K (mmol/L)	4.58 ± 0.40	4.67 ± 0.35	4.62 ± 0.20	4.90 ± 0.38	4.42 ± 0.24	4.47 ± 0.18
**Females**						
ALP (U/L)	67.1 ± 10.1	71.3 ± 10.2	63.3 ± 10.8	70.5 ± 11.5	57.0 ± 9.64	58.1 ± 8.21
ALT (U/L)	25.9 ± 4.20	28.2 ± 4.96	26.9 ± 4.21	27.1 ± 5.68	26.9 ± 6.45	25.7 ± 4.63
AST (U/L)	111 ± 14.1	117 ± 23.1	113 ± 24.2	102 ± 25.0	97.2 ± 19.3	87.5 ± 23.3
TBIL (mg/dL)	0.06 ± 0.02	0.07 ± 0.02	0.06 ± 0.03	0.06 ± 0.02	0.07 ± 0.01	0.07 ± 0.01
CHOL (mg/dL)	95.2 ± 13.1	93.3 ± 12.1	99.8 ± 17.8	139* ± 22.2	85.6 ± 21.9	89.1 ± 22.3
GLU (mg/dL)	139 ± 28.9	133 ± 20.6	133 ± 24.6	160 ± 38.5	103 ± 13.9	109 ± 18.2
UREA (mg/dL)	51.7 ± 9.56	52.3 ± 6.41	49.8 ± 6.39	48.6 ± 5.03	47.1 ± 10.1	43.5 ± 6.15
CREA (mg/dL)	0.38 ± 0.05	0.37 ± 0.03	0.38 ± 0.04	0.38 ± 0.02	0.44 ± 0.05	0.40 ± 0.04
PROT (g/dL)	5.51 ± 0.17	5.35 ± 0.15	5.50 ± 0.16	5.86* ± 0.17	4.74 ± 0.17	4.72 ± 0.41
ALB (g/dL)	3.82 ± 0.10	3.68 ± 0.08	3.78 ± 0.16	4.01* ± 0.12	3.36 ± 0.13	3.34 ± 0.28
GLO (g/dL)	1.69 ± 0.10	1.67 ± 0.09	1.72 ± 0.15	1.84* ± 0.09	1.38 ± 0.04	1.38 ± 0.15
A/G	2.26 ± 0.13	2.21 ± 0.11	2.20 ± 0.23	2.18 ± 0.08	2.46 ± 0.05	2.44 ± 0.15
Cl (mmol/L)	103 ± 1.59	102 ± 1.14	103 ± 1.58	100* ± 1.28	100 ± 1.68	98.2* ± 0.70
Ca (mmol/L)	2.47 ± 0.05	2.47 ± 0.05	2.51 ± 0.10	2.67* ± 0.12	2.17 ± 0.07	2.23 ± 0.19
Na (mmol/L)	141 ± 1.31	141 ± 0.87	142 ± 1.00	141 ± 1.15	137 ± 1.56	135 ± 1.51
K (mmol/L)	4.37 ± 0.35	4.09 ± 0.27	4.08 ± 0.20	4.21 ± 0.17	3.99 ± 0.24	4.08 ± 0.12

(* p-value < 0.05 compared to vehicle)

Alkaline Phosphatase (ALP), Alanine Aminotransferase (ALT), Aspartate Aminotransferase (AST), Albumin (ALB), Globulin (GLO), Albumin/Globulin ratio (A/G), Urea, Creatinine (CREA), Glucose (GLU), Total Bilirubin (TBIL), total Cholesterol (CHOL), total Protein (PROT), Sodium (Na), Potassium (K), Calcium (Ca) and Chloride (Cl).

Table S6: Mean clinical chemistry parameters in male and female Göttingen minipigs at the end of treatment and recovery.

	Main												Recovery					
**Dose (mg/kg)**	**0**			**18**			**82**			**409**			**0**			**409**		
**n**	**5**			**3**			**3**			**5**			**2**			**2**		
**Males**																		
ALP (U/L)	126	±	19.9	124	±	16.5	122	±	26.6	135	±	13.1	111	±	36.2	133	±	1.06
ALT (U/L)	41.3	±	3.12	38.0	±	4.51	45.6	±	4.45	42.2	±	3.29	31.0	±	0.49	36.3	±	2.12
AST (U/L)	22.4	±	3.20	27.6	±	8.98	27.5	±	5.42	30.9	±	14.2	19.2	±	3.46	19.7	±	3.46
TBIL (mg/dL)	0.04	±	0.01	0.04	±	0.01	0.02	±	0.02	0.03	±	0.02	0.02	±	0.00	0.03	±	0.01
CHOL (mg/dL)	61.2	±	12.6	58.7	±	12.0	51.8	±	10.1	63.9	±	10.6	61.8	±	3.18	49.1	±	1.70
TRIG (mg/dL)	38.0	±	12.9	25.2	±	1.56	35.1	±	7.53	20.4*	±	3.44	48.0	±	9.90	25.9	±	5.02
GLU (mg/dL)	99.8	±	14.3	93.3	±	6.52	91.6	±	6.35	88.7	±	7.18	90.4	±	6.58	93.3	±	14.8
UREA (mg/dL)	12.6	±	0.88	7.27*	±	1.01	13.1	±	1.36	9.94	±	3.15	10.7	±	0.64	11.9	±	1.77
CREA (mg/dL)	0.93	±	0.14	0.80	±	0.06	0.84	±	0.15	0.85	±	0.18	1.00	±	0.23	1.06	±	0.04
PROT (g/dL)	6.16	±	0.23	6.07	±	0.21	5.97	±	0.21	5.90	±	0.25	5.90	±	0.00	6.05	±	0.21
ALB (g/dL)	4.52	±	0.19	4.37	±	0.06	4.47	±	0.21	4.44	±	0.11	4.55	±	0.35	4.60	±	0.14
GLO (g/dL)	1.64	±	0.22	1.70	±	0.17	1.50	±	0.10	1.46	±	0.21	1.35	±	0.35	1.45	±	0.07
A/G	2.80	±	0.41	2.60	±	0.26	2.97	±	0.23	3.10	±	0.47	3.55	±	1.20	3.15	±	0.07
Cl (mmol/L)	99.7	±	1.34	97.7	±	1.95	99.5	±	2.10	97.3	±	2.13	101	±	0.21	96.2	±	0.07
Ca (mmol/L)	2.68	±	0.10	2.60	±	0.04	2.64	±	0.09	2.69	±	0.13	2.73	±	0.05	2.63	±	0.08
Na (mmol/L)	146	±	3.04	142	±	0.82	145	±	2.37	145	±	4.59	146	±	2.05	142	±	0.42
K (mmol/L)	5.14	±	0.73	4.52	±	0.61	5.23	±	0.78	5.70	±	1.04	5.46	±	0.67	4.76	±	0.95
**Females**																		
ALP (U/L)	99.2	±	13.3	98.7	±	10.0	101	±	21.5	127	±	79.4	136	±	9.83	129	±	6.51
ALT (U/L)	37.1	±	6.37	41.8	±	4.37	40.9	±	6.02	33.9	±	8.87	33.2	±	12.4	31.3	±	4.24
AST (U/L)	30.3	±	25.0	25.9	±	10.4	25.4	±	4.33	23.9	±	8.37	18.7	±	7.14	21.9	±	6.58
TBIL (mg/dL)	0.04	±	0.02	0.03	±	0.03	0.04	±	0.02	0.03	±	0.01	0.03	±	0.01	0.02	±	0.01
CHOL (mg/dL)	96.1	±	10.5	78.3	±	16.8	58.7*	±	8.32	77.3	±	14.6	87.1	±	12.5	80.5	±	8.77
TRIG (mg/dL)	43.6	±	14.1	45.5	±	24.5	38.9	±	7.89	33.9	±	13.5	53.3	±	18.8	49.6	±	15.3
GLU (mg/dL)	82.5	±	2.55	79.7	±	3.36	91.3	±	14.7	85.5	±	8.84	76.5	±	0.99	78.2	±	2.62
UREA (mg/dL)	14.4	±	2.63	12.7	±	4.38	14.9	±	5.58	13.3	±	3.46	11.3	±	3.54	15.7	±	5.59
CREA (mg/dL)	0.67	±	0.21	0.71	±	0.06	0.66	±	0.09	0.61	±	0.05	0.85	±	0.00	0.78	±	0.06
PROT (g/dL)	6.10	±	0.24	6.13	±	0.38	6.00	±	0.35	6.16	±	0.62	6.15	±	0.21	6.25	±	0.35
ALB (g/dL)	4.48	±	0.22	4.43	±	0.32	4.50	±	0.20	4.68	±	0.34	4.80	±	0.00	4.95	±	0.21
GLO (g/dL)	1.62	±	0.18	1.70	±	0.36	1.50	±	0.20	1.48	±	0.31	1.35	±	0.21	1.30	±	0.14
A/G	2.78	±	0.36	2.70	±	0.61	3.00	±	0.36	3.26	±	0.58	3.60	±	0.57	3.80	±	0.28
Cl (mmol/L)	99.7	±	1.59	99.0	±	1.23	98.9	±	0.91	97.5	±	1.10	102	±	1.13	101	±	0.07
Ca (mmol/L)	2.77	±	0.06	2.68	±	0.05	2.75	±	0.08	2.82	±	0.17	2.81	±	0.06	2.76	±	0.08
Na (mmol/L)	142	±	0.53	142	±	1.39	142	±	0.96	143	±	2.72	145	±	0.49	144	±	1.20
K (mmol/L)	4.71	±	0.26	4.63	±	0.31	4.50	±	0.26	4.78	±	1.00	5.08	±	1.56	4.29	±	0.13

(* p-value < 0.05 compared to vehicle)

Alkaline Phosphatase (ALP), Alanine Aminotransferase (ALT), Aspartate Aminotransferase (AST), Albumin (ALB), Globulin (GLO), Albumin/Globulin ratio (A/G), Urea, Creatinine (CREA), Glucose (GLU), Total Bilirubin (TBIL), total Cholesterol (CHOL), total Protein (PROT), Triglycerides (TRIG), Sodium (Na), Potassium (K), Calcium (Ca) and Chloride (Cl).

Table S7: Mean hematology parameters in male and female Sprague Dawley rats at the end of treatment and recovery.

	Main				Recovery	
**Dose (mg/kg)**	**0**	**27**	**136**	**682**	**0**	**682**
**n**	**10**	**10**	**10**	**9**	**5**	**5**
**Males**						
RBC (10^6/µL)	7.80 ± 0.28	7.68 ± 0.29	7.87 ± 0.29	8.03 ± 0.35	8.03 ± 0.13	7.73 ± 0.30
HCT (%)	44.4 ± 1.17	43.2 ± 1.32	44.9 ± 1.19	45.6 ± 1.75	44.4 ± 1.44	43.5 ± 1.04
HGB (g/dL)	14.8 ± 0.43	14.4 ± 0.42	14.8 ± 0.27	15.1 ± 0.58	14.8 ± 0.54	14.6 ± 0.27
MCV (fL)	57.0 ± 1.74	56.2 ± 0.61	57.0 ± 1.47	56.8 ± 1.08	55.3 ± 1.04	56.3 ± 1.47
MCH (pg)	19.0 ± 0.48	18.8 ± 0.25	18.9 ± 0.63	18.8 ± 0.37	18.4 ± 0.41	18.9 ± 0.54
MCHC (g/dL)	33.4 ± 0.57	33.5 ± 0.31	33.1 ± 0.52	33.1 ± 0.33	33.3 ± 0.46	33.6 ± 0.32
RET (10^9/µL)	187 ± 80.6	153 ± 23.5	176 ± 13.1	187 ± 16.3	168 ± 14.3	182 ± 30.2
WBC (10^3/µL)	8.71 ± 1.17	8.58 ± 2.25	8.70 ± 1.29	9.62 ± 1.81	9.16 ± 1.42	8.94 ± 0.87
NEU (10^3/µL)	0.88 ± 0.15	0.98 ± 0.29	0.99 ± 0.28	0.84 ± 0.27	0.99 ± 0.35	0.96 ± 0.15
LYM (10^3/µL)	7.20 ± 1.10	7.02 ± 1.99	7.16 ± 1.16	8.19 ± 1.49	7.61 ± 1.04	7.43 ± 0.77
MON (10^3/µL)	0.37 ± 0.10	0.36 ± 0.13	0.34 ± 0.11	0.35 ± 0.12	0.33 ± 0.13	0.33 ± 0.09
EOS (10^3/µL)	0.13 ± 0.04	0.11 ± 0.04	0.11 ± 0.03	0.09 ± 0.03	0.12 ± 0.02	0.11 ± 0.02
BAS (10^3/µL)	0.05 ± 0.01	0.04 ± 0.01	0.04 ± 0.02	0.05 ± 0.01	0.03 ± 0.01	0.04 ± 0.01
LUC (10^3/µL)	0.08 ± 0.02	0.08 ± 0.04	0.07 ± 0.02	0.09 ± 0.03	0.08 ± 0.02	0.07 ± 0.01
PLT (10^3/µL)	997 ± 76.7	1031 ± 101.3	1052 ± 93.0	1045 ± 72.3	969 ± 79.7	1029 ± 40.5
**Females**						
RBC (10^6/µL)	7.38 ± 0.21	7.42 ± 0.23	7.42 ± 0.19	7.44 ± 0.33	7.48 ± 0.19	7.49 ± 0.25
HCT (%)	41.0 ± 1.19	41.5 ± 1.23	41.9 ± 1.10	41.9 ± 1.41	41.6 ± 0.38	41.5 ± 1.83
HGB (g/dL)	13.8 ± 0.37	14.1 ± 0.42	14.1 ± 0.31	14.1 ± 0.50	14.2 ± 0.18	14.3 ± 0.49
MCV (fL)	55.5 ± 1.22	55.9 ± 0.98	56.5 ± 1.04	56.4 ± 1.28	55.6 ± 1.13	55.4 ± 0.88
MCH (pg)	18.8 ± 0.45	19.0 ± 0.39	19.0 ± 0.43	19.0 ± 0.40	19.0 ± 0.32	19.0 ± 0.22
MCHC (g/dL)	33.8 ± 0.43	34.0 ± 0.39	33.6 ± 0.60	33.6 ± 0.59	34.2 ± 0.30	34.4 ± 0.36
RET (10^9/µL)	154 ± 31.4	162 ± 26.3	193* ± 31.9	197* ± 33.8	131 ± 34.1	133 ± 20.5
WBC (10^3/µL)	5.49 ± 0.98	6.70 ± 1.56	5.30 ± 1.89	7.01 ± 1.47	5.89 ± 1.03	6.28 ± 0.78
NEU (10^3/µL)	0.51 ± 0.12	0.55 ± 0.16	0.54 ± 0.23	0.52 ± 0.10	0.53 ± 0.02	0.70 ± 0.17
LYM (10^3/µL)	4.63 ± 0.93	5.74 ± 1.42	4.41 ± 1.58	5.98 ± 1.32	5.03 ± 1.05	5.17 ± 0.72
MON (10^3/µL)	0.20 ± 0.08	0.24 ± 0.07	0.22 ± 0.08	0.32* ± 0.11	0.16 ± 0.03	0.23 ± 0.07
EOS (10^3/µL)	0.09 ± 0.02	0.09 ± 0.03	0.06* ± 0.02	0.09 ± 0.02	0.11 ± 0.02	0.11 ± 0.04
BAS (10^3/µL)	0.02 ± 0.01	0.03 ± 0.01	0.02 ± 0.01	0.03 ± 0.01	0.02 ± 0.01	0.02 ± 0.01
LUC (10^3/µL)	0.04 ± 0.02	0.06 ± 0.02	0.05 ± 0.02	0.06 ± 0.02	0.04 ± 0.02	0.05 ± 0.02
PLT (10^3/µL)	1076 ± 105	1036 ± 100	978 ± 80.0	1081 ± 90.8	1027 ± 70.3	1085 ± 66.0

(* p-value < 0.05 compared to vehicle)

Hematocrit (HCT), Hemoglobin (HGB), Red Blood Cell count (RBC), Reticulocyte count (RET), Mean Red Blood Cell Volume (MCV), Mean Corpuscular Hemoglobin (MCH), Mean Corpuscular Hemoglobin Concentration (MCHC), White Blood Cell count (WBC), Neutrophil count (NEU), Lymphocyte count (LYM), Eosinophil count (EOS), Basophil count (BAS), Monocyte count (MON), Large Unstained Cells (LUC) and Platelets (PLT).

Table S8: Mean hematology parameters in male and female Göttingen minipigs at the end of treatment and recovery.

	Main				Recovery	
**Dose (mg/kg)**	**0**	**16**	**82**	**409**	**0**	**409**
**n**	**5**	**3**	**3**	**5**	**2**	**2**
**Males**						
RBC (10^6/µL)	8.77 ± 1.08	8.75 ± 0.27	8.78 ± 0.54	8.81 ± 0.66	8.61 ± 0.26	8.46 ± 0.25
HCT (%)	46.0 ± 5.33	42.6 ± 1.16	46.6 ± 2.50	45.7 ± 4.34	44.6 ± 3.11	43.8 ± 0.28
HGB (g/dL)	14.9 ± 1.22	14.1 ± 0.17	15.5 ± 1.07	15.1 ± 1.02	14.8 ± 0.78	14.9 ± 0.00
MCV (fL)	52.4 ± 1.20	48.7 ± 1.07	53.2 ± 3.82	51.9 ± 2.04	51.8 ± 2.12	51.8 ± 1.84
MCH (pg)	17.0 ± 0.93	16.1 ± 0.55	17.7 ± 1.90	17.1 ± 0.67	17.2 ± 0.42	17.6 ± 0.57
MCHC (g/dL)	32.5 ± 1.36	33.1 ± 0.75	33.2 ± 1.56	33.0 ± 1.19	33.2 ± 0.49	34.0 ± 0.21
RET (10^9/µL)	85.5 ± 55.6	54.5 ± 29.5	72.8 ± 31.8	67.5 ± 42.0	84.2 ± 46.4	25.8 ± 10.1
WBC (10^3/µL)	10.9 ± 2.15	7.88 ± 0.32	8.46 ± 1.02	10.4 ± 1.98	9.09 ± 1.32	9.44 ± 1.75
NEU (10^3/µL)	3.84 ± 1.48	2.31 ± 0.76	3.24 ± 0.28	2.93 ± 0.59	2.45 ± 1.14	3.54 ± 0.39
LYM (10^3/µL)	6.20 ± 1.56	4.69 ± 0.71	4.57 ± 0.64	6.36 ± 1.43	5.79 ± 2.44	5.16 ± 1.00
MON (10^3/µL)	0.45 ± 0.12	0.48 ± 0.11	0.40 ± 0.13	0.52 ± 0.23	0.50 ± 0.01	0.46 ± 0.13
EOS (10^3/µL)	0.22 ± 0.10	0.29 ± 0.13	0.17 ± 0.02	0.43 ± 0.24	0.26 ± 0.00	0.20 ± 0.16
BAS (10^3/µL)	0.07 ± 0.04	0.05 ± 0.03	0.05 ± 0.02	0.10 ± 0.05	0.07 ± 0.01	0.04 ± 0.03
LUC (10^3/µL)	0.06 ± 0.04	0.05 ± 0.03	0.03 ± 0.03	0.08 ± 0.04	0.04 ± 0.01	0.05 ± 0.04
PLT (10^3/µL)	449 ± 111	367 ± 59.0	348 ± 44.2	387 ± 40.3	481 ± 53.7	472 ± 38.9
**Females**						
RBC (10^6/µL)	7.54 ± 0.79	7.66 ± 1.45	6.92 ± 0.77	7.81 ± 0.81	8.81 ± 1.97	8.75 ± 0.95
HCT (%)	38.6 ± 2.82	39.3 ± 5.48	39.1 ± 0.67	40.7 ± 5.09	44.4 ± 7.42	44.0 ± 4.60
HGB (g/dL)	13.3 ± 0.93	13.4 ± 1.69	13.7 ± 0.32	13.8 ± 1.45	15.0 ± 1.63	15.2 ± 1.48
MCV (fL)	51.5 ± 5.00	51.7 ± 4.56	56.9 ± 5.93	52.0 ± 1.96	50.7 ± 2.90	50.3 ± 0.28
MCH (pg)	17.8 ± 2.02	17.7 ± 1.86	19.9 ± 2.54	17.6 ± 0.43	17.2 ± 1.98	17.4 ± 0.21
MCHC (g/dL)	34.4 ± 0.85	34.2 ± 0.60	34.9 ± 0.85	33.9 ± 0.67	33.9 ± 2.05	34.5 ± 0.14
RET (10^9/µL)	21.3 ± 9.03	22.9 ± 11.0	20.7 ± 3.46	29.3 ± 18.4	32.4 ± 8.70	26.1 ± 5.66
WBC (10^3/µL)	8.88 ± 1.65	8.45 ± 1.86	8.95 ± 1.43	10.1 ± 3.04	7.81 ± 0.77	9.94 ± 4.96
NEU (10^3/µL)	2.31 ± 0.74	1.80 ± 0.46	2.33 ± 0.76	3.11 ± 2.39	1.48 ± 0.98	1.67 ± 0.89
LYM (10^3/µL)	5.48 ± 0.89	5.67 ± 1.63	5.52 ± 0.98	5.93 ± 2.10	5.63 ± 0.35	6.91 ± 3.20
MON (10^3/µL)	0.49 ± 0.23	0.51 ± 0.11	0.57 ± 0.08	0.56 ± 0.22	0.33 ± 0.09	0.66 ± 0.33
EOS (10^3/µL)	0.43 ± 0.23	0.31 ± 0.17	0.36 ± 0.38	0.26 ± 0.19	0.31 ± 0.04	0.55 ± 0.51
BAS (10^3/µL)	0.06 ± 0.02	0.05 ± 0.01	0.06 ± 0.01	0.08 ± 0.06	0.04 ± 0.01	0.07 ± 0.03
LUC (10^3/µL)	0.10 ± 0.09	0.11 ± 0.09	0.12 ± 0.01	0.12 ± 0.06	0.04 ± 0.00	0.09 ± 0.01
PLT (10^3/µL)	494 ± 51.9	416 ± 69.1	458 ± 55.4	358 ± 97.2	453 ± 67.9	463 ± 53.0

(* p-value < 0.05 compared to vehicle)

Hematocrit (HCT), Hemoglobin (HGB), Red Blood Cell count (RBC), Reticulocyte count (RET), Mean Red Blood Cell Volume (MCV), Mean Corpuscular Hemoglobin (MCH), Mean Corpuscular Hemoglobin Concentration (MCHC), White Blood Cell count (WBC), Neutrophil count (NEU), Lymphocyte count (LYM), Eosinophil count (EOS), Basophil count (BAS), Monocyte count (MON), Large Unstained Cells (LUC) and Platelets (PLT).

Table S9: Mean coagulation parameters in male and female Sprague Dawley rats at the end of treatment and recovery.

	Main				Recovery	
**Dose (mg/kg)**	**0**	**27**	**136**	**682**	**0**	**682**
**n**	**10**	**10**	**10**	**9**	**5**	**5**
**Males**						
PT (s)	24.4 ± 1.1	24.7 ± 0.7	23.8 ± 1.6	24.5 ± 0.5	25.1 ± 0.8	25.1 ± 1.7
APTT (s)	18.0 ± 1.3	16.5 ± 2.5	17.0 ± 2.0	18.1 ± 1.3	19.3 ± 0.7	18.1 ± 1.6
**Females**						
PT (s)	25.6 ± 1.0	26.3 ± 0.7	26.5 ± 0.8	25.2 ± 1.2	28.6 ± 0.2	28.1 ± 1.4
APTT (s)	16.5 ± 0.7	16.5 ± 0.7	16.6 ± 0.7	16.1 ± 1.3	16.5 ± 0.9	16.9 ± 1.1

(* p-value < 0.05 compared to vehicle)

Prothrombin Time (PT) and Activated Partial Thromboplastin Time (APTT)

Table S10: Mean coagulation parameters in male and female Göttingen minipigs at the end of treatment and recovery.

	Main				Recovery	
**Dose (mg/kg)**	**0**	**18**	**82**	**409**	**0**	**409**
**n**	**5**	**3**	**3**	**5**	**2**	**2**
**Males**						
PT (s)	14.1 ± 1.0	13.4 ± 0.6	14.4 ± 1.5	13.9 ± 0.7	14.4 ± 0.3	14.7 ± 0.6
APTT (s)	10.1 ± 0.6	11.1 ± 0.5	10.9 ± 0.7	10.0 ± 0.7	10.0 ± 0.7	10.4 ± 0.3
**Females**						
PT (s)	11.9 ± 0.8	12.2 ± 0.1	12.9 ± 0.5	14.0* ± 0.3	13.2 ± 0.0	13.7 ± 0.1
APTT (s)	10.2 ± 1.0	11.4 ± 2.0	10.1 ± 0.8	9.2 ± 1.2	10.5 ± 0.4	9.4 ± 0.1

(* p-value < 0.05 compared to vehicle)

Prothrombin Time (PT) and Activated Partial Thromboplastin Time (APTT)

Table S11: Mean organ weights in male and female Sprague Dawley rats at the end of treatment and recovery.

	Main				Recovery	
**Dose (mg/kg)**	**0**	**27**	**136**	**682**	**0**	**682**
**n**	**10**	**10**	**10**	**9**	**5**	**5**
**Males**						
Adrenal gland (g)	0.044 ± 0.010	0.051 ± 0.007	0.054* ± 0.005	0.051 ± 0.004	0.051 ± 0.006	0.054 ± 0.006
Brain (g)	1.798 ± 0.056	1.744 ± 0.087	1.838 ± 0.098	1.690* ± 0.063	1.760 ± 0.058	1.786 ± 0.064
Epididymis (g)	1.156 ± 0.110	1.168 ± 0.122	1.091 ± 0.143	1.201 ± 0.114	1.147 ± 0.081	1.329 ± 0.180
Heart (g)	1.175 ± 0.090	1.163 ± 0.110	1.246 ± 0.106	1.186 ± 0.090	1.170 ± 0.052	1.296 ± 0.115
Kidneys (g)	2.522 ± 0.182	2.446 ± 0.148	2.506 ± 0.133	2.563 ± 0.191	2.470 ± 0.152	2.766* ± 0.205
Liver (g)	10.17 ± 0.821	10.29 ± 0.759	11.22* ± 0.300	13.22* ± 1.058	9.740 ± 0.537	11.77* ± 0.942
Pituitary gland (g)	0.011 ± 0.002	0.010 ± 0.002	0.010 ± 0.002	0.011 ± 0.004	0.009 ± 0.002	0.011 ± 0.002
Prostate gland (g)	2.312 ± 0.228	2.202 ± 0.223	2.404 ± 0.230	2.301 ± 0.276	2.322 ± 0.168	2.848* ± 0.325
Spleen (g)	0.794 ± 0.094	0.754 ± 0.090	0.772 ± 0.053	0.724 ± 0.098	0.774 ± 0.080	0.787 ± 0.093
Testes (g)	3.600 ± 0.268	3.667 ± 0.145	3.441 ± 0.546	3.734 ± 0.222	3.480 ± 0.167	3.851* ± 0.250
Thymus (g)	0.501 ± 0.040	0.497 ± 0.082	0.471 ± 0.053	0.452 ± 0.038	0.396 ± 0.050	0.413 ± 0.058
Thyroid gland (g)	0.027 ± 0.004	0.027 ± 0.004	0.027 ± 0.005	0.030 ± 0.003	0.028 ± 0.002	0.025 ± 0.005
**Females**						
Adrenal gland (g)	0.062 ± 0.008	0.065 ± 0.006	0.063 ± 0.009	0.069 ± 0.010	0.064 ± 0.009	0.060 ± 0.007
Brain (g)	1.651 ± 0.112	1.669 ± 0.092	1.627 ± 0.095	1.608 ± 0.106	1.638 ± 0.103	1.646 ± 0.164
Heart (g)	0.863 ± 0.082	0.866 ± 0.085	0.867 ± 0.075	0.888 ± 0.096	0.904 ± 0.092	0.862 ± 0.037
Kidneys (g)	1.509 ± 0.137	1.523 ± 0.087	1.457 ± 0.084	1.542 ± 0.110	1.466 ± 0.178	1.526 ± 0.094
Liver (g)	6.357 ± 0.664	6.391 ± 0.666	6.403 ± 0.402	7.821* ± 1.086	6.138 ± 0.877	6.290 ± 0.450
Ovaries (g)	0.122 ± 0.021	0.125 ± 0.018	0.127 ± 0.018	0.128 ± 0.019	0.123 ± 0.015	0.113 ± 0.016
Pituitary gland (g)	0.013 ± 0.003	0.014 ± 0.003	0.013 ± 0.002	0.011 ± 0.003	0.014 ± 0.002	0.012 ± 0.003
Spleen (g)	0.599 ± 0.059	0.615 ± 0.077	0.572 ± 0.054	0.642 ± 0.062	0.600 ± 0.103	0.538 ± 0.038
Thymus (g)	0.331 ± 0.038	0.306 ± 0.056	0.346 ± 0.037	0.366 ± 0.062	0.307 ± 0.056	0.313 ± 0.045
Thyroid gland (g)	0.017 ± 0.002	0.018 ± 0.002	0.016 ± 0.003	0.018 ± 0.003	0.027 ± 0.002	0.024 ± 0.005
Uterus (g)	0.653 ± 0.335	0.671 ± 0.355	0.786 ± 0.311	0.717 ± 0.374	0.736 ± 0.283	0.682 ± 0.343

(* p-value < 0.05 compared to vehicle)

Differences in adrenal, brain, prostate and testes organ weight between the vehicle group and treatment groups were without macroscopic and microscopic findings. In addition, differences were within range of spontaneous findings and therefore considered incidental and unrelated to treatment.

Table S12: Mean organ weights in male and female Göttingen minipigs at the end of treatment and recovery.

	Main				Recovery	
**Dose (mg/kg)**	**0**	**16**	**82**	**409**	**0**	**409**
**n**	**3**	**3**	**3**	**3**	**2**	**2**
**Males**						
Adrenal gland (g)	1.435 ± 0.14	1.778 ± 0.27	1.657 ± 0.16	1.783 ± 0.26	1.422 ± 0.18	2.038 ± 0.31
Brain (g)	64.34 ± 2.52	64.02 ± 3.84	58.63 ± 5.01	62.05 ± 4.47	63.68 ± 8.70	63.20 ± 0.54
Epididymis (g)	31.34 ± 12.9	26.74 ± 2.54	38.75 ± 7.21	32.66 ± 9.20	30.56 ± 5.64	29.42 ± 1.99
Gall bladder (g)	2.141 ± 0.74	2.438 ± 1.21	2.208 ± 0.23	4.106 ± 2.11	1.669 ± [x]	3.761 ± 0.59
Heart (g)	105.6 ± 5.33	119.4 ± 14.0	109.4 ± 6.72	110.7 ± 16.0	107.0 ± 4.93	114.5 ± 0.33
Kidneys (g)	103.8 ± 2.51	86.43* ± 5.01	97.07 ± 7.41	96.96 ± 7.52	102.8 ± 18.9	90.50 ± 15.1
Liver (g)	466.1 ± 75.9	376.7 ± 17.5	424.8 ± 25.8	450.6 ± 15.7	483.9 ± 17.5	381.9 ± 28.1
Pituitary gland (g)	0.148 ± 0.02	0.134 ± 0.01	0.157 ± 0.02	0.132 ± 0.03	0.139 ± 0.03	0.116 ± [x]
Spleen (g)	69.33 ± 28.5	40.73 ± 9.60	53.85 ± 13.0	58.68 ± 24.8	49.14 ± 2.76	60.45 ± 16.2
Testes (g)	41.63 ± 7.80	31.74 ± 8.44	55.96 ± 9.02	43.30 ± 0.65	33.63 ± 3.21	36.68 ± 16.7
Thymus (g)	19.38 ± 4.35	21.04 ± 5.16	19.89 ± 3.51	26.79 ± 14.1	24.07 ± 8.85	24.89 ± 4.78
Thyroid gland (g)	1.274 ± 0.41	1.426 ± 0.14	1.590 ± 0.35	1.594 ± 0.42	1.805 ± 0.36	1.197 ± 0.08
**Females**						
Adrenal gland (g)	1.705 ± 0.43	1.473 ± 0.39	1.705 ± 0.23	1.949 ± 0.49	2.138 ± 0.12	2.167 ± 0.69
Brain (g)	66.49 ± 4.94	59.59 ± 6.42	65.43 ± 5.33	57.76 ± 1.86	66.15 ± 1.12	72.15 ± 1.68
Gall bladder (g)	3.743 ± 2.06	4.475 ± 1.94	3.761 ± 0.88	3.825 ± 3.45	2.447 ± 0.28	3.421 ± 0.01
Heart (g)	97.87 ± 13.1	92.68 ± 12.8	94.61 ± 12.2	102.0 ± 2.66	95.02 ± 11.9	102.4 ± 15.2
Kidneys (g)	104.6 ± 20.3	96.51 ± 6.44	84.29 ± 6.90	102.0 ± 20.3	82.00 ± 1.29	101.4 ± 1.27
Liver (g)	431.3 ± 57.6	405.8 ± 21.2	409.7 ± 35.6	481.1 ± 45.6	395.3 ± 42.4	401.3 ± 126
Ovaries (g)	3.742 ± 0.82	3.989 ± 2.10	2.762 ± 1.20	3.181 ± 1.56	3.365 ± 0.85	3.988 ± 0.74
Pituitary gland (g)	0.130 ± 0.03	0.126 ± 0.02	0.151 ± 0.02	0.110 ± 0.01	0.113 ± 0.00	0.161 ± 0.01
Spleen (g)	76.83 ± 37.1	86.77 ± 35.1	98.79 ± 58.5	55.18 ± 12.0	53.04 ± 14.8	73.85 ± 30.0
Thymus (g)	23.61 ± 2.70	19.41 ± 1.50	26.24 ± 3.40	21.75 ± 8.66	18.00 ± 0.93	17.43 ± 1.96
Thyroid gland (g)	1.391 ± 0.13	1.342 ± 0.24	1.199 ± 0.13	1.792 ± 0.41	1.773 ± 0.41	1.862 ± 0.07
Uterus (g)	275.3 ± 64.3	231.1 ± 94.5	184.8 ± 21.5	178.3 ± 51.7	189.1 ± 66.3	257.5 ± 97.8

(* p-value < 0.05 compared to vehicle; [X] organs were missing at necropsy)

Differences in organ weight between the vehicle group and treatment groups were without macroscopic and microscopic findings. In addition, differences were within range of spontaneous findings and therefore considered incidental and unrelated to treatment.
